# New Smart Polymeric Materials with Optical Sensing Properties: Determination of Antimicrobial Properties Using Photodynamic Therapy

**DOI:** 10.3390/polym18202478

**Published:** 2026-10-10

**Authors:** Tomás Valdés, Isabel Cáceres, Oscar G. Marambio, Julio Sánchez, Christian Erick Palavecino, Guadalupe del C. Pizarro

**Affiliations:** 1 Departamento de Química, Facultad de Ciencias Naturales, Matemáticas y Medio Ambiente, Universidad Tecnológica Metropolitana, J. P. Alessandri 1242, Ñuñoa, Santiago 7800002, Chile; tvaldes@utem.cl (T.V.); isabel.caceresi@utem.cl (I.C.); omarambi@utem.cl (O.G.M.); 2 Departamento de Química Orgánica, Facultad de Química y de Farmacia, Pontificia Universidad Católica de Chile, Santiago 7820436, Chile; julio.sanchez@uc.cl; 3 Laboratorio de Microbiología Molecular y Fotodinámica, Centro de Ciencias Médicas Aplicadas, Facultad de Medicina y Ciencias de la Salud, Universidad Central de Chile, Lord Cochrane 418, Santiago 8330546, Chile; christian.palavecino@ucentral.cl

**Keywords:** spiropyran, quantum dots, antimicrobial photodynamic therapy, polymer

## Abstract

Antimicrobial resistance has intensified the search for non-antibiotic strategies activated by external stimuli, including antimicrobial photodynamic therapy. In this work, a hybrid system based on spiropyran-functionalized poly(maleic anhydride-alt-acrylic acid), P(MAn-*alt*-AA)-SP, and CdTe@ZnS quantum dots incorporated at 5 wt.% was prepared and characterized. Structural, thermal, morphological, and optical properties were evaluated by ^1^H NMR, FTIR, TGA, UV–Vis spectroscopy, fluorescence spectroscopy, TEM, and XRD. Irradiation at 365 nm induced the conversion of spiropyran SP to merocyanine MC, evidenced by the appearance of an absorption band near 559 nm and a visible color change. CdTe@ZnS quantum dots exhibited an average diameter of 3.29 ± 0.94 nm, while the hybrid system displayed a broad emission band centered at approximately 635 nm, whose intensity depended on molecular oxygen. Antibacterial assays against methicillin-sensitive *Staphylococcus aureus* showed increased activity after irradiation, with the hybrid formulation exhibiting the highest mean inhibition among the evaluated systems. Overall, the results are consistent with an interaction between the photoactive polymer and quantum dots and consistent with the participation of reactive oxygen species (ROS) in photodynamic processes, highlighting the potential of this hybrid material for the development of light-responsive antimicrobial platforms. These findings support future antimicrobial coatings and surface applications.

## 1. Introduction

Antimicrobial resistance (AMR) has emerged as a major global public health threat in the 21st century because bacteria increasingly evade conventional treatments, progressively limiting therapeutic options for previously treatable infections. Recent statistical models estimated that approximately 4.95 million deaths were associated with resistant infections in 2019, including 1.27 million directly attributable to AMR [1]. Furthermore, projections suggest that this number could reach nearly 10 million annual deaths by 2050 if effective control strategies are not implemented [2]. In response, the World Health Organization established a priority pathogen list based on resistance level and public health relevance [3]. Among these, carbapenem-resistant Klebsiella pneumoniae, third-generation cephalosporin-resistant Escherichia coli, fluoroquinolone-resistant Salmonella Typhi, methicillin-resistant *Staphylococcus aureus* (MRSA), and methicillin-sensitive *Staphylococcus aureus* (MSSA) are associated with high morbidity and difficult clinical management. MRSA is strongly linked to skin and soft tissue infections, as well as severe conditions such as bacteremia, sepsis, and endocarditis, often resulting in poorer clinical outcomes compared to susceptible strains [4]. Despite advances in infection control and therapeutic strategies, the persistence of these pathogens highlights the limitations of current approaches against resistant bacterial infections.

Even with the extensive development of antimicrobial agents over recent decades, the effectiveness of many antibiotic treatments has been compromised by the remarkable adaptive capacity of bacteria under the selective pressure exerted by these drugs [5]. Consequently, microorganisms have developed resistance mechanisms such as enzymatic degradation, membrane permeability alterations, and active efflux systems [6]. In addition, bacteria such as MRSA may acquire resistance through horizontal gene transfer, where a single genetic acquisition event can generate fully antibiotic-resistant strains. Moreover, the development of new antibiotic classes has remained limited for decades, failing to keep pace with bacterial evolution and highlighting the need for alternative therapeutic strategies [7].

Therefore, developing non-antibiotic antimicrobial therapies has become essential. Among the emerging strategies proposed to combat multidrug-resistant (MDR) bacteria, antimicrobial photodynamic therapy (PDT) has attracted increasing attention [8]. Photodynamic therapy is a photochemical treatment based on the combined use of light and a photoresponsive compound known as a photosensitizer (PS) [9]. The origins of this strategy date back to the late 19th century, with one of the earliest reported applications attributed to Niels Ryberg Finsen, who received the Nobel Prize in 1903 for the treatment of lupus vulgaris using ultraviolet (UV) light [10]. The photochemical process involved in PDT begins when the PS absorbs a photon at an appropriate wavelength, promoting the transition from the ground state (S_0_) to an excited singlet state (S_1_). Subsequently, the PS may return to the ground state through fluorescence emission or undergo intersystem crossing (ISC) toward a triplet excited state (T_1_), characterized by longer lifetimes and a greater ability to interact with molecular oxygen [11,12]. The interaction between the triplet excited state and molecular oxygen can generate reactive oxygen species (ROS) pathway Type I and Type II photodynamic mechanisms [13]. In the Type I mechanism, electron or hydrogen transfer generates free radicals such as hydroxyl radicals (OH•), superoxide anions (O_2_•^−^), and hydrogen peroxide (H_2_O_2_). In contrast, the Type II mechanism involves direct energy transfer to molecular oxygen, primarily promoting the formation of singlet oxygen (^1^O_2_) [14]. These highly reactive species induce oxidative damage to bacterial organic structures, including membranes, proteins, and nucleic acids, ultimately leading to bacterial cell death [15].

Despite the potential of PDT, its efficiency largely depends on the properties of the photosensitizer and its interaction with the biological environment [16]. In this regard, free photosensitizers may have limitations, including low solubility, nonspecific distribution, rapid elimination, and insufficient accumulation at the target site, which can reduce photodynamic performance and increase the need for higher doses [17]. These challenges have driven the development of structured platforms capable of protecting, transporting, and localizing photoactive species, thereby promoting a more controlled and efficient photodynamic response.

These limitations of free photosensitizers, including low solubility, nonspecific distribution, rapid elimination, and insufficient accumulation at the target site, can directly affect photodynamic performance by reducing the availability of the active excited state and limiting effective interaction with molecular oxygen. For this reason, the design of polymeric or nanostructured platforms capable of stabilizing photoactive species, controlling their spatial distribution, and promoting efficient energy transfer processes has become a relevant strategy to improve the applicability of PDT [18].

In this context, photoactive polymers have emerged as promising platforms for designing more stable, controllable, and functional photodynamic systems [19]. Unlike free photosensitizers, polymeric matrices allow the incorporation of photoresponsive units within a macromolecular structure, promoting their immobilization, stability, and spatial control [20]. Among the most widely studied photoactive groups, spiropyrans stand out due to their ability to undergo a reversible transformation between a closed, colorless, and less polar form known as spiropyran SP, and an open, colored, zwitterionic form known as merocyanine MC, induced by UV irradiation or other external stimuli [21]. This structural conversion modifies the optical, electronic, and polarity properties of the system, enabling the development of materials with photochromic response, coordination ability, and potential interaction with nanostructured species [22].

Within this approach, copolymers derived from maleic anhydride (Man) and acrylic acid (AA) represent attractive polymeric matrices for the design of functional materials with potential biomedical applications. Although the free monomers may exhibit high chemical reactivity, their incorporation into macromolecular structures enables the formation of materials containing anhydride and carboxylic acid groups available for chemical modification, hydrogen bonding, ionic interactions, and coordination processes [23]. Acrylic-acid-derived polymers have been widely studied as hydrophilic, environmentally responsive, and potentially biocompatible platforms due to their swelling capacity, pH-responsive behavior, and usefulness in controlled drug delivery, nanomedicine, tissue engineering, and antimicrobial applications [24,25]. Likewise, P(MAn-*alt*-AA) copolymers have been reported to behave as responsive systems whose properties can be modulated by pH, ionic strength, and post-polymerization functionalization [26]. Moreover, maleic-anhydride-based copolymers have shown versatility in biomedical and antimicrobial applications [27,28] due to their functionalizable structure, tunable hydrophilic/hydrophobic balance, and ability to form conjugates or active matrices [29]. Therefore, the combination of MAn- and AA-derived units enables the design of a polar, chemically modifiable, and potentially biocompatible matrix suitable for integrating photoactive units and promoting interactions with nanostructured species.

In this functional matrix, incorporating spiropyran units is particularly attractive due to the versatility of these organic photochromic compounds for stimuli-responsive materials [30]. Beyond their photochromic response, the merocyanine MC form exhibits a polar, zwitterionic structure that can engage in recognition, chelation, or coordination with metal ions or semiconductor surfaces [31]. This behavior has been widely exploited in SP-based chemosensors for the colorimetric and fluorescent detection of metal ions and anions, in which complexation shifts the SP/MC equilibrium and the system’s spectroscopic response [32]. Moreover, in hybrid platforms with nanoparticles, spiropyran derivatives have been shown to reversibly modulate the photoluminescence of quantum dots through energy-transfer mechanisms, supporting their potential as organic units capable of linking molecular response with fluorescent nanocomposites [33].

Because SP derivatives can reversibly modulate the photoluminescence of quantum dots through energy-transfer processes, semiconductor QDs are a particularly suitable nanostructured counterpart for designing hybrid photoactive platforms [34]. Their optical properties are strongly governed by quantum confinement, which allows absorption and emission phenomena to be modulated at the nanoscale [35]. In addition, the surface chemistry of quantum dots (QDs) plays a decisive role in their colloidal stability, photoluminescence, and interaction with molecules or functional matrices [36]. In particular, CdTe@ZnS QDs combine a CdTe semiconductor core, responsible for size-dependent absorption and photoluminescence, with a wider-bandgap ZnS shell, whose function is to passivate surface defects, decrease non-radiative recombination processes, and improve the optical stability of the nanocrystal [37]. Complementarily, studies in analogous semiconductor core–shell systems have demonstrated that ZnS coating can significantly increase photoluminescence quantum yield by passivating non-radiative surface recombination sites [38]. Moreover, in aqueous CdTe-based QDs, architectures with an external ZnS layer have been reported to markedly reduce cytotoxicity associated with Cd^2+^ ion release, highlighting the protective role of surface coatings in systems with potential biological applications [39]. On the other hand, semiconductor QDs have been investigated as light-responsive nanomaterials that can participate in photodynamic processes via energy transfer to nearby photoactive molecules [40]. Additionally, recent reviews have highlighted their potential to mediate processes associated with reactive oxygen species generation under irradiation, further expanding interest in their use in photodynamic platforms [41]. Therefore, the incorporation of CdTe@ZnS QDs into P(MAn-*alt*-AA)-SP allows the projection of a hybrid architecture in which the polymeric matrix, the photochromic unit, and the semiconductor nanocomponent may act in an integrated manner.

Although the integration of semiconductor QDs into photoactive polymeric matrices offers a promising route for developing hybrid photodynamic materials, an important gap remains in understanding the interfacial phenomena that govern their behavior. This gap is particularly relevant because the optical performance of QDs critically depends on their synthesis, surface chemistry, colloidal stability, and energy-transfer mechanisms, all of which condition their interaction with molecules, functional matrices, and biological systems [42]. In this regard, studies based on self-assembled QD–biomolecule assemblies have demonstrated that Förster resonance energy transfer (FRET) can modulate QD photoluminescence, but its efficiency is strongly conditioned by the proximity between the semiconductor donor core and the molecular acceptor, as well as by the spatial organization of the assembly [43]. Likewise, photoswitchable QDs have been described in which a polymer coating functionalized with photochromic units enables reversible modulation of nanocrystal emission through photochromic Förster resonance energy transfer (pcFRET) processes, demonstrating that the localization of the photochromic acceptor within the QD surface environment is determinant for the photoluminescent response [44]. Addressing this gap is essential for establishing chemical design criteria for photoactive hybrid materials with enhanced optical control and potential application in antimicrobial photodynamic strategies.

Based on this gap, the present work proposes the design and characterization of a hybrid photoactive platform based on P(MAn-*alt*-AA)-SP/CdTe@ZnS, conceived as a chemical response to the challenge of integrating, within a single material, a functional polymeric matrix, a photochromic unit, and a semiconductor nanocomponent with controllable optical response. This approach is supported by previous studies demonstrating that hybrid polymeric films can exhibit morphological and optical properties modulated by the incorporation of nanocomponents and by the organization of the polymeric matrix [45]. Likewise, microporous hybrid films obtained from amphiphilic copolymers and surface-coated with ZnS nanoparticles have shown that the structuring methodology and the presence of the inorganic phase enable the construction of nano-organized polymeric surfaces with functional properties associated with the material architecture [46]. More specifically, photoswitchable polymeric systems containing spiropyran units, in the presence of ZnS nanoparticles, have shown that UV irradiation can induce changes associated with SP/MC interconversion, thereby modifying the optical and morphological response of the system [47]. From an antimicrobial perspective, photo antimicrobial polymers based on cationic matrices functionalized with photosensitizers have been developed, showing the ability to induce bacterial inactivation under irradiation against relevant pathogens, including *Staphylococcus aureus* [48]. Similarly, electrospun polymeric fibers containing photosensitizers have been proposed as light-activatable platforms for antibiotic-free infection control [49]. Consequently, the incorporation of CdTe@ZnS QDs into P(MAn-*alt*-AA)-SP is proposed as an advance toward a hybrid architecture in which the SP/MC photochromic response, QD photoluminescence, and polymeric microenvironment can be correlated within a single photoactive platform. Thus, this work seeks to establish relationships between structure, interface, and photophysical response that contribute to the rational design of polymer–nanoparticle materials with potential application in antimicrobial photodynamic strategies.

To address this approach, the present study combines structural, thermal, morphological, and photophysical characterization with a preliminary biological evaluation. The hybrid system was examined using complementary techniques, including ^1^H NMR, FTIR, TGA, UV–Vis spectroscopy, fluorescence spectroscopy, TEM, and XRD, thereby enabling assessment of its chemical structure, photochromic behavior, thermal stability, nanostructural features, and optical response within a single analytical framework. Finally, the antimicrobial performance against MSSA was preliminarily assessed under dark and irradiated conditions through minimum inhibitory concentration (MIC) determination and Kirby–Bauer agar diffusion assays, allowing the relationship between the photoactive character of the hybrid material and its potential antibacterial response to be explored.

## 2. Materials and Methods

### 2.1. Reagents

All chemicals were of analytical grade and used without further purification unless otherwise specified. Maleic anhydride (MAn), acrylic acid (AA), tetrahydrofuran (THF), and benzoyl peroxide (BPO, 99.98%) were purchased from Sigma-Aldrich (St. Louis, MO, USA). Diethyl ether, magnesium sulfate, sodium carbonate, hydrochloric acid, chloroform, acetone, dimethyl sulfoxide (DMSO), methanol, benzene, triethylamine, ethanol, concentrated sulfuric acid, acetonitrile, zinc chloride (ZnCl_2_), and thioacetamide (TAA) were obtained from Merck (Darmstadt, Germany). 2,3,3-Trimethylindolenine, 2-bromoethanol, 2-butanone, and 2-hydroxy-5-nitrobenzaldehyde were purchased from Sigma-Aldrich (St. Louis, MO, USA).

For QDs synthesis, cadmium chloride (CdCl_2_, 99%), cadmium acetate dihydrate [(CH_3_COO)_2_Cd·2H_2_O], 3-mercaptopropionic acid (MPA, 97%), tellurium powder (Te, 99%), and sodium borohydride (NaBH_4_, 98%) were purchased from Sigma-Aldrich Química Ltda. (Santiago, Chile). Sodium sulfide (Na_2_S), zinc acetate dihydrate [(CH_3_COO)_2_Zn·2H_2_O], potassium dihydrogen phosphate (KH_2_PO_4_), sodium hydroxide (NaOH), anhydrous sodium sulfite (Na_2_SO_3_), and potassium hydroxide (KOH) were obtained from Merck (Darmstadt, Germany). All reagents were used as received without additional purification.

### 2.2. Measurements

The chemical structure of the spiropyran-functionalized monomer and the resulting P(MAn-*alt*-AA)-SP copolymer was characterized by proton nuclear magnetic resonance ^1^H NMR spectroscopy using a Bruker Avance III HD 400 MHz spectrometer (Bruker, Karlsruhe, Germany). The ^1^H NMR spectra were recorded using CDCl_3_ as the solvent. Attenuated total reflectance Fourier-transform infrared (ATR-FTIR) spectra were obtained using a Shimadzu IRSpirit spectrometer (Shimadzu, Kyoto, Japan) equipped with a QATR-S accessory. The samples were placed directly on the diamond crystal and analyzed over the range of 4000–500 cm^−1^ at a resolution of 1 cm^−1^.

X-ray diffraction (XRD) measurements were performed using a Bruker D2 Phaser diffractometer (Bruker, Karlsruhe, Germany) with Co Kα radiation (λ = 1.7902 Å). The diffraction patterns were recorded over a 2θ range of 10–90°, using a step size of 0.05° and a counting time of 5 s per step.

The morphology and particle-size distribution of the CdTe@ZnS quantum dots were examined by transmission electron microscopy (TEM) using a Hitachi HT7700 transmission electron microscope (Hitachi, Tokyo, Japan). Particle-size analysis was performed from the TEM micrographs by measuring individual nanoparticles and fitting the resulting size distribution with a Gaussian function.

UV–Vis absorption spectra were recorded using a PerkinElmer Lambda 35 spectrophotometer (PerkinElmer, Waltham, MA, USA) at a spectral resolution of 1 nm. Measurements were performed using quartz cuvettes with a 1 cm optical path length. Fluorescence excitation and emission spectra were obtained using a Shimadzu RF-5301PC spectrofluorophotometer (Shimadzu, Kyoto, Japan) at a spectral resolution of 1 nm. Excitation spectra were acquired by scanning the excitation wavelength while monitoring fluorescence at a fixed emission wavelength, whereas emission spectra were acquired using a fixed excitation wavelength while scanning the emission wavelength. For the excitation spectrum of P(MAn-*alt*-AA)-SP, the scan range was selected to exclude the region affected by solvent/background interference and to maintain spectral separation from the monitored emission wavelength, thereby minimizing contributions from scattered excitation light. Emission measurements under ambient and inert N_2_ atmospheres were performed using the same instrumental settings. The spectra were evaluated as experimentally recorded, without independent normalization, spectral deconvolution, post-acquisition mathematical fitting, or extrapolation beyond the experimentally acquired ranges. Photoluminescence measurements under ambient and inert N_2_ atmospheres were used to evaluate the influence of molecular oxygen on the emission behavior of P(MAn-*alt*-AA)-SP, CdTe@ZnS quantum dots, and the P(MAn-*alt*-AA)-SP/CdTe@ZnS hybrid system.

Thermogravimetric analysis (TGA) was performed using a METTLER TOLEDO STARe thermal analysis system (Mettler-Toledo, Greifensee, Switzerland) under a nitrogen atmosphere. A 100 mg sample was placed in a 70 μL alumina crucible and heated from 25 to 800 °C at a constant heating rate of 25 °C min^−1^.

### 2.3. Synthesis of Indolinium Precursor

The indolinium precursor, 1-(2-hydroxyethyl)-2,3,3-trimethylindolenine bromide, was synthesized following a previously reported procedure with slight modifications [50]. In a pre-evacuated Schlenk tube under an inert argon atmosphere, 2,3,3-trimethylindolenine (4.0 mL, 25 mmol), 2-bromoethanol (1.8 mL, 25 mmol), and 2-butanone (3.15 mL, 35 mmol) were introduced. The reaction mixture was subjected to freeze–pump–thaw cycles using liquid nitrogen to remove dissolved oxygen. This process was repeated several times until complete degassing was achieved. Subsequently, the reaction mixture was heated in an oil bath at 140 °C under constant stirring for 10 h. After completion, the system was cooled to room temperature, and the resulting pink solid was collected by filtration. The crude product was purified by Soxhlet extraction using benzene for 24 h until the solvent became colorless, indicating the removal of residual impurities. The purified product was then dried and used in subsequent reactions.

### 2.4. Synthesis of Spiropyran SP

The SP was prepared by condensation of the previously obtained indolinium precursor with 2-hydroxy-5-nitrobenzaldehyde, as described in the literature [51]. In a 250 mL three-neck round-bottom flask, 1-(2-hydroxyethyl)-2,3,3-trimethylindolenine bromide (2.00 g) and 2-hydroxy-5-nitrobenzaldehyde (1.20 g) were combined. Triethylamine (4 mL) was added as a base, followed by ethanol (20 mL) as solvent. The system was equipped with a condenser and a thermometer, and the reaction mixture was heated in an oil bath at 78 °C under constant stirring for 4 h. After completion of the reaction, the mixture was cooled to room temperature, and the resulting solid was collected by filtration, washed with cold ethanol, and dried under vacuum. The final spiropyran was obtained as a colored solid and stored in the dark to prevent premature photoisomerization.

### 2.5. Synthesis of AA-SP Monomer by Fischer Esterification

The AA-SP monomer was synthesized by Fischer esterification of the hydroxyl-functionalized SP with acrylic acid, following classical esterification procedures [52]. In a 10 mL round-bottom flask, acrylic acid (1.0 mL, 15 mmol) was used as both reagent and solvent in excess. SP (400 mg, 1.1 mmol) was added, followed by a catalytic amount of concentrated sulfuric acid. The reaction mixture was heated in a boiling water bath for 60 min with intermittent manual agitation. After completion of the reaction, the mixture was cooled to room temperature. Chloroform (2.0 mL) was added to extract the organic phase, followed by distilled water (1.0 mL) to promote phase separation. The aqueous and organic phases were separated, and the organic phase was collected for subsequent polymerization.

### 2.6. Synthesis of the P(MAn-alt-AA)-SP Copolymer

The copolymer was synthesized via free-radical polymerization of the AA-SP monomer and MAn under an inert atmosphere. The use of a 1:1 monomer feed ratio and BPO as radical initiator was selected according to previously reported procedures for alternating maleic anhydride-based copolymer systems [53]. In addition, the incorporation of spiropyran units into maleic anhydride-derived copolymers has been previously reported, supporting the design of the present photoactive polymeric system [54]. In a Schlenk reactor, AA-SP (200 mg, 0.49 mmol) and MAn (48 mg, 0.49 mmol) were dissolved in acetonitrile (3.0 mL). BPO, (1.19 mg, 4.9 μmol, 0.50 mol%) was then added as radical initiator. Once complete dissolution was achieved, the reaction mixture was subjected to three freeze–pump–thaw cycles using liquid nitrogen to remove dissolved oxygen and establish an inert nitrogen atmosphere. After degassing, the reactor was allowed to return to room temperature and then placed in a thermostatic glycerin bath at 80 °C for 3 h. Upon completion of the reaction, the resulting solution was transferred to a pre-weighed beaker, covered with perforated aluminum foil, and left to evaporate at room temperature for 24 h. The resulting polymer was then stored in a vacuum desiccator until further use.

### 2.7. Aqueous Synthesis of CdTe Quantum Dot QD Core

CdTe QDs were synthesized via an aqueous colloidal method using a freshly prepared NaHTe precursor. The telluride precursor (NaHTe) was prepared by reducing tellurium with sodium borohydride under nitrogen atmosphere, as reported elsewhere [55]. The NaHTe solution was subsequently reacted with an aqueous solution of CdCl_2_ previously degassed under nitrogen atmosphere. The reaction was carried out at pH 9 in the presence of mercaptocarboxylic acids acting as stabilizing ligands. The mixture was subjected to reflux conditions to promote controlled nucleation and growth of CdTe nanocrystals. Under these conditions, nanoparticles with estimated sizes ranging from 2.8 to 4.0 nm were obtained [56].

### 2.8. Aqueous Synthesis of CdTe@ZnS Quantum Dot QD Core–Shell

The CdTe@ZnS core–shell quantum dots were synthesized through a modified aqueous approach based on previously reported methods for the preparation of water-soluble CdSe@ZnS nanocrystals [57], with adaptations to accommodate CdTe cores. The CdTe cores used for ZnS shell formation retained the 3-mercaptopropionic acid (MPA) stabilizing ligands introduced during their aqueous synthesis, whose hydrophilic carboxylate groups favor the subsequent dispersion of the CdTe@ZnS QDs in water.

Following the synthesis of CdTe core quantum dots, the reaction mixture was allowed to cool to room temperature under continuous stirring. The pH was subsequently adjusted to 7.5–8.0 to ensure optimal conditions for metal ion coordination and to minimize the formation of undesired zinc hydroxide species. An aqueous zinc precursor solution was then prepared by dissolving 5.0 × 10^−5^ mol of ZnCl_2_ in 5 mL of Milli-Q water. This solution was added dropwise to the flask containing the CdTe cores under vigorous stirring. The system was maintained under stirring for 30 min to facilitate the adsorption of Zn^2+^ ions onto the surface of the nanocrystals, promoting a homogeneous distribution prior to shell formation. After this adsorption step, the reaction temperature was increased to 70 °C. In parallel, a sulfur precursor solution was prepared by dissolving 1.5 × 10^−4^ mol of thioacetamide (TAA) in 5 mL of Milli-Q water. The TAA solution was then added dropwise to the reaction mixture under continuous stirring, allowing a controlled release of sulfide ions and promoting uniform ZnS shell growth around the CdTe cores. Under these conditions, thioacetamide undergoes thermal decomposition, gradually releasing S^2−^ ions in situ. These sulfide ions react with the pre-adsorbed Zn^2+^ species on the CdTe surface, leading to the controlled nucleation and growth of a ZnS shell around the CdTe cores. This stepwise process is critical to ensure uniform shell coverage and to avoid independent ZnS nucleation in solution. The reaction was maintained at 70 °C for 1 h to allow complete shell formation. The growth of the ZnS shell contributes to the effective passivation of surface trap states, resulting in enhanced photoluminescence properties and improved chemical stability of the quantum dots. Finally, the CdTe@ZnS core–shell quantum dots were purified by precipitation using an antisolvent (e.g., ethanol or isopropanol), followed by centrifugation and redispersion in Milli-Q water for further characterization.

### 2.9. Preparation of the Polymer–Quantum Dot QD Hybrid System

The polymer–quantum dot hybrid system was prepared by incorporating CdTe@ZnS quantum dots into the photoactive P(MAn-*alt*-AA)-SP copolymer matrix, following previously reported approaches for integrating semiconductor nanocrystals into polymeric environments to produce optically functional hybrid materials [58,59]. Briefly, P(MAn-*alt*-AA)-SP was dissolved in chloroform under magnetic stirring until a homogeneous polymer solution was obtained. An aqueous dispersion of CdTe@ZnS quantum dots was subsequently added at 5 wt.% relative to the dry mass of the copolymer, corresponding to 5 mg of CdTe@ZnS for every 100 mg of P(MAn-*alt*-AA)-SP. The resulting biphasic mixture was sonicated in an ultrasonic bath for 10 min to promote the dispersion of the quantum dots throughout the polymer-containing system and was subsequently maintained under magnetic stirring. Chloroform was then allowed to evaporate, while the CdTe@ZnS quantum dots remained suspended in the residual aqueous phase. The water was subsequently evaporated until a dry hybrid material was obtained. All preparation steps were performed under protection from ambient light to minimize unintended photoisomerization of the spiropyran units. The resulting material was designated P(MAn-*alt*-AA)-SP/CdTe@ZnS and used for subsequent photophysical characterization and antibacterial evaluation.

### 2.10. Antibacterial Assays

The antimicrobial activity of the different formulations was evaluated against MSSA using a direct contact assay performed in 96-well microplates containing solid Mueller–Hinton agar, following previously described methodologies for the evaluation of photoactive materials with antimicrobial activity [60,61,62]. The formulations evaluated included the photochromic agent spiropyran SP, the photochromic agent incorporated into the copolymer P(MAn-*alt*-AA)-SP, free CdTe@ZnS nanoparticles, and nanoparticles incorporated into the composite matrix P(MAn-*alt*-AA)-SP/CdTe@ZnS. The bacterial suspension was prepared in Mueller–Hinton broth and adjusted to an initial concentration of 1 × 10^7^ colony-forming units (CFU)·mL^−1^, corresponding to an optical density at 600 nm (OD600) of 0.4–0.6. Serial dilutions of the different formulations were prepared to evaluate concentration-dependent antimicrobial activity. In addition, the antimicrobial efficacy of the materials was assessed against bacterial inocula ranging from 10^7^ to 10^0^ CFU·mL^−1^.

For formulations containing SP, the bacterial suspension was combined with the corresponding test formulation at the evaluated concentration directly in the wells containing solid Mueller–Hinton agar. The samples were then irradiated with 365 nm UV light for 10 min, followed by 525 nm visible green light for 10 min, to induce the reversible photoisomerization of the photochromic agent in the presence of MSSA, according to previously described protocols for photodynamic materials [63,64]. The green light irradiation was performed using a Kessil PR160-525 nm lamp (DiCon Fiberoptics, Richmond, CA, USA), with a nominal peak emission wavelength of 525 nm and an average fluence rate of 11.29 mW/cm^2^, measured at a working distance of 13 cm using a S120VC photodetector (Thorlabs, Newton, NJ, USA). The average fluence rate was calculated from triplicate measurements at five positions across the irradiation area. The corresponding light fluence was 6.77 J/cm^2^ for the 10 min exposure. The UV light irradiation was performed using a Spectroline ENF-240C lamp (Spectronics Corporation, Melville, NY, USA), with a nominal peak emission wavelength of 365 nm and an average fluence rate 300 μW/cm^2^. After irradiation, the microplates were incubated at 37 °C for 16 h. At the end of the incubation period, antimicrobial activity was determined by counting the bacterial colonies grown in each well and comparing them with the growth and sterility controls. The minimum inhibitory concentration (MIC) was defined as the lowest concentration of each formulation required to completely inhibit MSSA colony formation [65,66]. The results were expressed as colony-forming units (CFU)/mL, and all experiments were performed in triplicate.

### 2.11. Agar Diffusion Assay (Kirby–Bauer)

The antimicrobial activity of the different formulations was also evaluated using the Kirby–Bauer agar diffusion method, following the standardized disk diffusion procedure originally described by Bauer et al., and the general recommendations for antimicrobial susceptibility testing [67,68]. Briefly, a suspension of methicillin-sensitive *Staphylococcus aureus* MSSA was adjusted to a turbidity equivalent to the 0.5 McFarland standard and uniformly inoculated onto Mueller–Hinton agar plates using a sterile cotton swab to obtain homogeneous bacterial growth over the entire agar surface [68,69].

Sterile paper disks of 6 mm in diameter were impregnated with the different formulations evaluated: blank, copolymer P(MAn-*alt*-AA), photochromic agent SP, photochromic agent incorporated into the copolymer P(MAn-*alt*-AA)-SP, free CdTe@ZnS nanoparticles, and the composite matrix P(MAn-*alt*-AA)-SP/CdTe@ZnS. The disks were carefully placed onto the surface of the previously inoculated Mueller–Hinton agar plates. Photoactivation was performed directly on the plates by irradiation with 365 nm UV light for 10 min, followed by 525 nm visible light for 10 min. The plates were then incubated at 37 °C for 16 h. All assays were performed in triplicate (*n* = 3).

After incubation, antimicrobial activity was determined by measuring the diameter of the inhibition zones formed around each disk, as commonly used for the in vitro evaluation of antimicrobial activity [61,69]. The inhibition zone diameters were recorded in millimeters and expressed as the mean ± standard deviation (SD) of three independent experiments. This assay was used as a complementary method to compare the inhibitory activity of the different formulations against MSSA.

## 3. Results

### 3.1. Characterization of the Spiropyran-Functionalized Acrylic Monomer (AA-SP)

The successful esterification between AA and SP was evaluated by FTIR spectroscopy. Figure 1A shows the comparative spectra of pristine AA and the functionalized monomer AA-SP. The spectrum of AA exhibits a broad O–H stretching band associated with the carboxylic acid group, together with the characteristic carbonyl and vinyl-group vibrations. After functionalization, significant changes are observed in the AA-SP spectrum. The broad O–H stretching band observed in AA is no longer detected in AA-SP, indicating consumption of the carboxylic acid functionality during esterification. In addition, the C=O stretching band shifts from approximately 1691 cm^−1^ in AA to approximately 1724 cm^−1^ in AA-SP, consistent with the conversion of the carboxylic acid group into an ester functionality. Changes are also observed in the C=C stretching region, with the band detected at approximately 1613 cm^−1^ in AA appearing at approximately 1622 cm^−1^ in AA-SP. Since vibrations from both the acrylic vinyl group and the aromatic structure of the spiropyran moiety may contribute to this region, this band alone is not considered diagnostic of spiropyran incorporation. In addition, several new absorption features are observed in the fingerprint region of the AA-SP spectrum (approximately 1500–700 cm^−1^), which are consistent with vibrations associated with the aromatic, C–N, C–O, and nitro-containing functionalities introduced by the spiropyran moiety. The aliphatic C–H stretching bands observed in the 2961–2890 cm^−1^ region remain present after functionalization. Complementary structural characterization of the AA-SP monomer was performed by ^1^H NMR and two-dimensional ^1^H–COSY spectroscopy in CDCl_3_ (Figures S1 and S2, Supplementary Material). The ^1^H NMR spectrum shows resonances associated with both the spiropyran and acrylate moieties. In particular, signals at approximately δ 6.38, 6.06, and 5.83 ppm are consistent with the vinyl protons of the acrylate group, indicating preservation of the polymerizable C=C bond after functionalization. Signals in the methylene region, around δ 4.4 and 2.7 ppm, are consistent with the proton environments of the –N–CH_2_–CH_2_–O– linkage. In addition, no broad resonance is observed in the approximately δ 11–12 ppm region characteristic of a carboxylic acid proton, which is consistent with ester formation. The 2D COSY spectrum provides complementary proton–proton correlations supporting these assignments. Taken together, the FTIR, ^1^H NMR, and 2D COSY results provide complementary evidence for the successful formation of the AA-SP monomer.

### 3.2. Structural Characterization of P(MAn-alt-AA)-SP

#### 3.2.1. Copolymer by ^1^H NMR

The structure of the P(MAn-*alt*-AA)-SP copolymer was confirmed by ^1^H NMR spectroscopy Figure 2. The ^1^H NMR (δ, ppm, CDCl_3_) exhibit the following signals: at 8.00 (CHCl_3_); at 7.22 (1H, t, Hb); at 7.15 (1H, d, Hd); at 7.05 (1H, m, Hi); at 6.89 (1H, d, He); at 6.70 (1H, d, Hf); at 6.64 (1H, d, Hc); at 6.07 (1H, d, Ha); at 6.07 (2H, m, Hk, –CH_2_O–); at 4.28 (2H, m, Hj, –CH_2_N<); at 3.45–0.50 (aliphatic region, see Figure 2).

The spectrum exhibits characteristic signals corresponding to aromatic, methylene, and aliphatic proton environments, consistent with the proposed polymer structure. Signals in the aromatic region (δ 6.50–8.00 ppm) are attributed to the spiropyran moiety, while broad resonances in the aliphatic region (δ 0.50–2.50 ppm) correspond to the polymer backbone. Notably, the signal at δ 4.28 ppm is assigned to methylene protons adjacent to the ester linkage, confirming the successful incorporation of the spiropyran-functionalized monomer into the polymer structure. The signal at 5.8 ppm is attributed to possible coexistence of the SP and MC. The observed chemical shifts and signal distribution support the formation of the P(MAn-*alt*-AA)-SP copolymer.

#### 3.2.2. Copolymer by FT-IR

The formation of the P(MAn-*alt*-AA)-SP copolymer was evaluated by FTIR spectroscopy. Figure 3 shows the comparative spectra of maleic anhydride MAn, the spiropyran-functionalized monomer AA-SP, and the resulting copolymer.

The FTIR spectrum of MAn exhibits characteristic bands at 1850 and 1720 cm^−1^, corresponding to the asymmetric and symmetric stretching vibrations of the anhydride carbonyl groups (C=O), along with a band at 831 cm^−1^ attributed to C–O–C stretching of the anhydride functionality. Additionally, a band at 1631 cm^−1^ is assigned to C=C stretching of the alkene group. The AA-SP spectrum shows characteristic bands in the range of 2961–2890 cm^−1^ associated with aliphatic C–H stretching, together with a carbonyl absorption around 1720 cm^−1^. An additional absorption at approximately 1020 cm^−1^ is also observed in the functionalized monomer. After copolymerization, the FTIR spectrum of P(MAn-*alt*-AA)-SP retains the characteristic carbonyl bands of the anhydride groups at 1850 and 1720 cm^−1^, as well as the C–O–C band at 831 cm^−1^. Importantly, the disappearance of the alkene C=C band at ~1631 cm^−1^ indicates the consumption of vinyl groups during the polymerization process. Furthermore, the persistence of the aliphatic C–H stretching bands (3120–2890 cm^−1^), together with the characteristic carbonyl and C–O–C absorptions observed in the copolymer spectrum, is consistent with the formation of the resulting polymer structure. These spectral changes provide strong evidence for the successful formation of the P(MAn-*alt*-AA)-SP copolymer.

#### 3.2.3. Copolymer by TGA

The thermogram of the P(MAn-*alt*-AA)-SP copolymer exhibits four distinct thermal decomposition temperatures (TDTs). The derivative thermogravimetry (DTG) curve represents the first derivative of the mass-loss curve with respect to temperature and provides information on the rate of mass loss during heating. Four main events can be distinguished in the DTG profile, corresponding to the thermal decomposition stages observed in the TGA curve. The first TDT is observed up to approximately 100 °C and is attributed to the loss of residual solvent. The second TDT, at 173 °C, is directly associated with cleavage of the ester bond, thereby releasing the photochromic moiety incorporated into the polymer chain. The third TDT, observed at 227 °C, corresponds to the decomposition of the hydrocarbon chains of both the polymer matrix and the photochromic agent. Finally, the fourth TDT, at 440 °C, is related to the thermal degradation of the MAn units, leaving a residue of approximately 11% of the initial mass. Therefore, the DTG curve complements the TGA by allowing these four thermal events to be distinguished more clearly. Based on these results, the polymer can be considered thermally stable for applications below 170 °C, as decomposition processes begin above this temperature; therefore, its use should be limited to conditions below this threshold (see Figure 4).

### 3.3. Photophysical Analysis of P(MAn-alt-AA)-SP and CdTe@ZnS Quantum Dots

The photochromic behavior of the P(MAn-*alt*-AA)-SP copolymer was evaluated by UV–Vis spectroscopy in chloroform solution (0.2 mg/mL) for 5 min of irradiation at 365 nm. A separate irradiation-time-dependent kinetic study was not performed to independently determine the photostationary state. Figure 5 shows the absorption spectrum.

Upon irradiation at 365 nm, a new absorption band appears in the visible region of the spectrum in Figure 5A at approximately 559 nm, corresponding to the MC form. This band is attributed to the formation of the MC isomer, resulting from the cleavage of the C–O spiro bond and subsequent ring opening. The emergence of this new band is consistent with the formation of the merocyanine MC form, whose ring-opened structure exhibits a more extended π-conjugated system and absorption in the visible region. This transition confirms the preservation of the spiropyran moiety’s photochromic properties after copolymerization. Additionally, a visible color change was observed during UV irradiation, consistent with the SP → MC transformation. Figure 5 illustrates the evolution of this process. Initially, the polymer is in the SP form (see Figure 5A), characterized by a closed-ring, non-colored structure. The solution initially exhibited the characteristic appearance of the predominantly closed SP form Figure 5B. After UV irradiation, the development of a purple coloration was consistent with the formation of an MC-enriched state Figure 5C. The photoluminescence properties of the P(MAn-*alt*-AA)-SP copolymer were evaluated to establish its reference photophysical response prior to its interaction with quantum dots. Figure 6 shows the fluorescence excitation spectrum of the P(MAn-*alt*-AA)-SP copolymer (black), together with the fluorescence emission spectra recorded under ambient conditions (blue) and under an inert N_2_ atmosphere (red). The emission spectra were recorded using an excitation wavelength of 390 nm. Under ambient conditions, the copolymer exhibits an emission maximum centered at approximately 620 nm, whereas under an inert N_2_ atmosphere, the emission maximum is observed at approximately 612 nm with a higher fluorescence intensity.

The observed red emission is attributed to the merocyanine MC form of the SP moiety, whose open-ring structure gives rise to an extended π-conjugated system capable of absorbing and emitting in the visible region. As shown in Figure 6, the emission profile varies depending on the atmospheric conditions used during the measurement. In particular, the lower fluorescence intensity recorded under ambient conditions is consistent with oxygen-mediated quenching processes, in which molecular oxygen interacts with the excited states of the emissive species and promotes additional non-radiative deactivation pathways. By contrast, removal of oxygen under an N_2_ atmosphere reduces these quenching processes, resulting in a higher emission intensity.

The slight shift in the emission maximum from approximately 612 nm under an inert atmosphere to 620 nm under ambient conditions may also reflect changes in the polarity and electronic microenvironment surrounding the merocyanine units within the polymer matrix. Such changes could modify the stabilization of the emissive state without substantially altering the overall photochromic behavior of the copolymer. Therefore, the results demonstrate that the fluorescence response of P(MAn-*alt*-AA)-SP is sensitive to the atmospheric conditions used during the measurement. Although these steady-state fluorescence data do not directly demonstrate triplet-state population or reactive oxygen species generation, the oxygen-dependent variation in emission supports the occurrence of interactions between molecular oxygen and the excited states of the photoactive polymer. This behavior provides a relevant photophysical basis for the subsequent evaluation of the material in photodynamic antimicrobial applications.

In the case of core–shell quantum dots composed of a cadmium telluride (CdTe) core and a zinc sulfide (ZnS) shell, Figure 7A shows an absorption peak around 470 nm. The emission spectra corresponding to Figure 7B, show that, under both inert and ambient atmospheres, the emission maximum remains approximately constant at 517 nm. However, emission intensity varies with the atmosphere.

The observed decrease in emission intensity under ambient conditions can be attributed to oxygen-induced quenching processes. Molecular oxygen, which exists in a triplet ground state (^3^O_2_), can efficiently interact with excited states through collisional energy transfer, promoting non-radiative decay pathways. This interaction is commonly associated with the population of triplet excited states via intersystem crossing (ISC), enabling subsequent energy transfer to oxygen and the potential formation of reactive oxygen species (ROS), such as singlet oxygen (^1^O_2_), as widely reported in organic chromophores [70]. This oxygen-dependent quenching is consistent with an interaction between molecular oxygen and the excited states of the quantum dots. However, steady-state fluorescence measurements alone do not directly demonstrate triplet-state population or ROS generation.

### 3.4. Physical Characterization of CdTe@ZnS Quantum Dots and Hybrid Material

TEM analysis confirms the formation of well-dispersed quantum dots with predominantly spherical morphology and good spatial distribution across the substrate (see Figure 8A). TEM analysis of the P(MAn-*alt*-AA)-SP/CdTe@ZnS hybrid material Figure 8B reveals a high-contrast inorganic domain associated with the lower-contrast polymeric matrix, providing morphological evidence of the incorporation of CdTe@ZnS QDs into the hybrid system. The original, unprocessed TEM micrographs, are provided in the Supplementary Materials. These observations confirm the incorporation of the QDs within the hybrid material. A statistical analysis was performed by measuring 506 individual nanoparticles from the TEM micrographs. The corresponding size distribution histogram was fitted with a Gaussian function, resulting in an average particle diameter of 3.29 ± 0.94 nm (see Figure 8D). The relatively narrow size dispersion indicates a controlled nucleation and growth process, consistent with the formation of quasi-monodisperse nanocrystals. This behavior highlights the role of synthesis conditions in regulating particle growth and achieving nanoscale size uniformity. In addition, the relatively small particle size is expected to enhance quantum confinement effects, which are crucial for tuning the optical properties of semiconductor nanocrystals. The good dispersion and absence of large aggregates further suggest that the surface ligands effectively prevent particle coalescence, contributing to the colloidal stability of the system. These structural features are particularly relevant for optoelectronic applications, where uniformity in size and morphology directly impacts the reproducibility and efficiency of the photophysical response.

The crystalline structure of the synthesized CdTe@ZnS quantum dots was analyzed by X-ray diffraction (XRD), as shown in Figure 9. The diffraction pattern provides insight into the structural organization of the nanocrystals, enabling evaluation of their crystallographic phase as well as the structural effects associated with the formation of the core–shell architecture. In nanoscale systems, the diffraction features are typically broadened due to the reduced crystallite size, while variations in peak position and profile may reflect interfacial strain and lattice mismatch between the core and shell components. In this context, the analysis of the diffraction pattern is particularly relevant to assess the degree of structural coherence and the influence of ZnS shell growth on the CdTe core.

The diffraction pattern exhibits three main peaks located at 2θ = 28.46°, 47.64°, and 56.31°, which are indexed to the (111), (220), and (311) crystallographic planes of the cubic zinc blende structure. Notably, the position of these peaks is consistent with previously reported CdTe@ZnS systems, where the growth of a ZnS shell over a CdTe core induces a shift in the diffraction peaks toward those characteristics of ZnS. This behavior is attributed to lattice mismatch and strain effects between the core and shell materials [71]. These observations are consistent with the formation of CdTe@ZnS core–shell quantum dots.

### 3.5. Photophysical Analysis of the P(MAn-*alt*-AA)-SP/CdTe@ZnS Hybrid System

The photoluminescent behavior of the P(MAn-*alt*-AA)-SP/CdTe@ZnS hybrid system was evaluated by fluorescence spectroscopy using an excitation wavelength of 390 nm and a slit bandwidth of 10 nm. The hybrid material, prepared by incorporating 5 wt.% CdTe@ZnS quantum dots relative to the dry mass of the copolymer, was analyzed at a copolymer concentration of 0.2 mg/mL under ambient and inert N_2_ atmospheres.

Figure 10 shows the fluorescence emission spectra obtained for the P(MAn-*alt*-AA)-SP/CdTe@ZnS hybrid system under ambient and inert N_2_ atmospheres. Under both conditions, the hybrid material exhibited a broad emission band with a maximum located at approximately 635 nm. No appreciable shift in the position of the emission maximum was observed between the two atmospheric conditions. However, a marked difference in fluorescence intensity was detected, with the emission recorded under the inert N_2_ atmosphere being considerably higher than that obtained under ambient conditions.

The preservation of the emission maximum position indicates that the predominant emissive state of the hybrid system remains unchanged under both conditions. In contrast, the variation in intensity demonstrates that the photoluminescent response of the material depends on the atmosphere used during the measurement. These results confirm that incorporating CdTe@ZnS into the P(MAn-*alt*-AA)-SP matrix produces a hybrid system with characteristic emission in the red region of the visible spectrum and an intensity sensitive to the presence of ambient oxygen.

### 3.6. Antibacterial Activity

The antimicrobial activity of the different formulations is presented in Figure 11. Overall, all formulations containing photoactive compounds exhibited a concentration-dependent antibacterial response, characterized by a progressive reduction in bacterial viability as the concentration increased. Free CdTe@ZnS nanoparticles Figure 11A began to inhibit bacterial growth at concentrations of 0.02 mg/mL, reaching bacterial counts close to the detection limit at 0.15 mg/mL. This behavior is consistent with previous reports describing the antimicrobial activity of semiconductor quantum dots, which is mainly attributed to the generation of reactive oxygen species (ROS), leading to oxidative damage of bacterial membranes, proteins, and nucleic acids, as well as alterations in membrane permeability [72].

The incorporation of CdTe@ZnS nanoparticles into the spiropyran-functionalized copolymer P(MAn-*alt*-AA)-SP/CdTe@ZnS markedly enhanced antibacterial efficacy Figure 11B, yielding significant reductions in bacterial viability at lower concentrations than those required for free nanoparticles. Evaluations were conducted using a twofold serial dilution series starting from an initial concentration of 300 μg/mL of CdTe@ZnS nanoparticles (300, 150, 75, 38, and 19 μg/mL) while maintaining a fixed spiropyran concentration of 20 mg/mL. Likewise, incorporating spiropyran into the copolymer backbone P(MAn-*alt*-AA)-SP substantially improved its antimicrobial performance compared with free spiropyran Figure 11C, suggesting that the polymeric matrix promotes a more homogeneous distribution of the photoactive compound and enhances its interaction with bacterial cells. Among all tested formulations, the hybrid system comprising the copolymer, spiropyran, and CdTe@ZnS nanoparticles Figure 11D exhibited the strongest antimicrobial activity, producing a pronounced reduction in bacterial viability at lower concentrations than those required for the individual components. For the complete hybrid matrix, evaluations were carried out using twofold serial dilutions starting from an initial functionalized spiropyran concentration of 20 mg/mL (20, 10, 5, 2.5, and 1.25 mg/mL) maintaining a fixed CdTe@ZnS nanoparticle concentration of 20 µg/mL. The combined formulation exhibited enhanced antibacterial activity under the tested conditions, which may be associated with interactions between the photochromic spiropyran and semiconductor nanoparticles. These interactions could potentially influence electron transfer processes and ROS generation upon photoactivation; however, further experiments are required to elucidate the underlying mechanisms responsible for the observed antibacterial response. Similar findings have been reported for hybrid photoactive nanomaterials designed for antimicrobial photodynamic applications, in which the combination of photoswitchable molecules and semiconductor nanoparticles significantly enhances bacterial inactivation against *Staphylococcus aureus* by increasing ROS production and photodynamic efficiency [73,74].

### 3.7. Antimicrobial Activity Evaluated by the Kirby–Bauer Agar Diffusion Assay

The results obtained using the agar diffusion assay (Table 1) confirmed the antimicrobial activity observed in the direct contact experiments. Neither the negative control nor the P(MAn-*alt*-AA) copolymer produced inhibition zones under either irradiation or dark conditions, confirming that the polymeric matrix itself lacks intrinsic antibacterial activity. In contrast, free CdTe@ZnS nanoparticles and free spiropyran exhibited moderate antimicrobial activity that increased markedly after sequential irradiation with 365 nm UV and 525 nm visible light, indicating that light activation is crucial for enhancing their antibacterial effects. All assays were performed in triplicate (*n* = 3).

The P(MAn-*alt*-AA)-SP and P(MAn-*alt*-AA)-SP/CdTe@ZnS formulations produced the largest inhibition zones under both irradiated and non-irradiated conditions, reaching diameters of 14.5 ± 1.33 mm and 14.9 ± 0.47 mm, respectively, after photoactivation. In addition to increasing the inhibition zone diameter shown in Figure 12, irradiation reduced experimental variability, indicating improved reproducibility of the photoactive system. These findings indicate that the simultaneous incorporation of spiropyran and CdTe@ZnS nanoparticles into the polymeric matrix is associated with an enhanced antibacterial response under the tested conditions. This effect may be related to improved spatial organization of the photoactive components and their potential interaction at the polymer–quantum-dot interface; however, the specific contribution of CdTe@ZnS to the antibacterial activity cannot be established from the agar diffusion assay alone. Similar results have been reported for photoactive nanomaterials used in antimicrobial photodynamic therapy, in which the combination of photosensitizers and semiconductor nanoparticles significantly enhances antibacterial activity against *S. aureus* compared with the individual components [75,76].

## 4. Discussion

The results obtained by FTIR and ^1^H NMR support the formation of the proposed photoactive polymeric platform. The disappearance of the signals associated with the vinyl groups after polymerization, together with the preservation of the bands corresponding to the anhydride groups and the aromatic spiropyran fraction, is consistent with the incorporation of the AA-SP monomer into the P(MAn-*alt*-AA)-SP structure. Complementarily, the aromatic and methylene signals observed by ^1^H NMR confirm the presence of the spiropyran unit attached to the copolymer. Thermogravimetric analysis showed that the main degradation processes begin above approximately 170 °C, indicating that the matrix exhibits adequate thermal stability for the irradiation and biological evaluation conditions employed in this study.

Functionalization of the monomer and its subsequent incorporation into the copolymer did not suppress the photochemical response of SP. After irradiation at 365 nm, an absorption band appeared at approximately 559 nm, accompanied by the characteristic color change associated with the SP → MC transformation. This result demonstrates that the photoactive unit retains its ability to undergo ring opening and form the conjugated merocyanine structure after polymerization. The red emission observed for P(MAn-*alt*-AA)-SP, with maxima near 612 and 620 nm under inert and ambient atmospheres, respectively, is also consistent with the presence of emissive states associated with the open MC form.

TEM analysis revealed predominantly spherical quantum dots with an average diameter of 3.29 ± 0.94 nm and no extensive aggregation in the analyzed micrographs. The relatively narrow particle-size distribution is consistent with a controlled nucleation and growth process. Furthermore, the XRD reflections assigned to the (111), (220), and (311) planes correspond to a cubic zinc-blende structure and support the formation of the CdTe@ZnS architecture. These features are relevant because nanocrystal size, dispersion, and surface passivation influence both their photoluminescence and their interaction with the polymeric matrix.

The incorporation of 5 wt.% CdTe@ZnS into P(MAn-*alt*-AA)-SP produced a hybrid system with a broad emission band centered at approximately 635 nm. This maximum is shifted toward longer wavelengths relative to the copolymer, whose emission was located between 612 and 620 nm, and is clearly separated from the emission of the free quantum dots at approximately 517 nm. The absence of a clearly resolved independent band near 517 nm and the shift in the dominant emission indicate that the hybrid does not behave as a simple superposition of its individual components. Instead, the results are consistent with the formation of an organic–inorganic interface capable of modifying the electronic microenvironment of the photoactive units.

From a chemical perspective, this interaction can be explained by coordination of the merocyanine form with available metal centers at the CdTe@ZnS surface. Ring opening generates a zwitterionic structure containing a phenolic oxygen with high electron density, which can act as a donor site toward surface Zn^2+^ centers. Complex formation between the open form of spiropyrans and Zn^2+^ has previously been confirmed by ^1^H NMR, showing conversion of the closed form into a trans-merocyanine–Zn^2+^ complex [77]. Furthermore, a recent study using CdSe/ZnS quantum dots demonstrated experimentally and through DFT calculations that the anionic oxygen of merocyanine exhibits a high affinity for surface Zn^2+^ centers. In that system, the calculated binding energy for MC–Zn^2+^ was more favorable than that of the carboxylate ligand initially present at the quantum-dot surface [78]. Although these calculations were performed for a related CdSe/ZnS system, they provide theoretical support for the proposed interaction between merocyanine and surface Zn^2+^ centers in the present CdTe@ZnS system.

This interpretation accounts for the shift in the emission maximum to 635 nm and the absence of a clearly distinguishable independent quantum-dot contribution. Coordination not only maintains both components in close spatial proximity but also modifies the electronic environment of merocyanine and generates a collective photophysical response. The observed effect can therefore be related to the formation of a coordinated interface in which the polymeric matrix organizes the photoactive units, merocyanine binds to surface metal centers, and the quantum dots participate in the modulation of the excited states.

Rigidification of a merocyanine does not necessarily imply increased ROS formation, as fluorescence, internal conversion, intersystem crossing, and electron transfer constitute competing excited-state deactivation pathways. However, in the present system, the combination of interfacial coordination, modified emission, and pronounced oxygen-induced quenching indicates that a fraction of the excited-state population remains available to interact with O_2_. From a photodynamic perspective, this interaction may promote Type I mechanisms via electron transfer and radical-species formation, or Type II mechanisms via energy transfer and singlet-oxygen generation.

The biological assays showed a concentration-dependent reduction in bacterial viability for the photoactive formulations. Free CdTe@ZnS quantum dots began to inhibit bacterial growth at 0.02 mg/mL and produced bacterial counts close to the detection limit at 0.15 mg/mL. This result agrees with studies showing that photoexcited quantum dots can alter bacterial redox states through photogenerated charge carriers, producing activity against different bacterial isolates [79]. CdTe quantum dots have also been shown to associate bacterial surfaces, impair cellular antioxidant systems, and induce oxidative damage to proteins and lipids through an ROS-mediated pathway [80].

Interestingly, the agar diffusion assay revealed detectable antibacterial activity of P(MAn-*alt*-AA)-SP under non-irradiated conditions (10.0 ± 0.67 mm), whereas neither free spiropyran nor the unfunctionalized P(MAn-*alt*-AA) copolymer produced inhibition zones in the dark. This observation suggests that incorporation of spiropyran into the polymeric matrix may modify the local physicochemical environment, accessibility, or interaction of the photochromic moiety with bacterial cells, resulting in an antibacterial effect that is not detectable for the individual components. However, the present experiments were not designed to determine the mechanism underlying this dark activity, and therefore a specific molecular mechanism cannot be established from the current data. Notably, incorporation of CdTe@ZnS into P(MAn-*alt*-AA)-SP produced only a small increase in the inhibition zone under non-irradiated conditions (from 10.0 ± 0.67 to 10.67 ± 1.11 mm) and a similarly limited change after irradiation (from 14.5 ± 1.33 to 14.9 ± 0.47 mm). Thus, within the agar diffusion assay, the addition of CdTe@ZnS did not result in a pronounced additional antibacterial effect. This relatively small difference may also reflect limitations inherent to diffusion-based antimicrobial assays, in which the size, physicochemical properties, and diffusivity of nanomaterials can influence the formation of inhibition zones and potentially lead to an underestimation of their activity when their mobility through agar is limited [81,82]. This limitation may be particularly relevant to the present hybrid system, in which CdTe@ZnS nanoparticles are incorporated within a polymeric matrix, potentially restricting their release or accessibility to bacterial cells. These findings indicate that the simultaneous incorporation of spiropyran and CdTe@ZnS nanoparticles into the polymeric matrix is associated with an enhanced antibacterial response under the tested conditions. However, the relatively small difference between P(MAn-*alt*-AA)-SP and P(MAn-*alt*-AA)-SP/CdTe@ZnS in the agar diffusion assay does not allow the specific contribution of the quantum dots to be independently established. The observed response may be influenced by the organization, accessibility, and diffusion of the photoactive components within the polymeric matrix.

## 5. Conclusions

This work reports the preparation of a photoactive hybrid system based on P(MAn-*alt*-AA)-SP and CdTe@ZnS quantum dots. Structural characterization confirmed the incorporation of the spiropyran-functionalized monomer into the copolymer, while UV–Vis spectroscopy demonstrated that the SP–MC photoisomerization was preserved after polymerization. CdTe@ZnS quantum dots exhibited a predominantly spherical morphology, an average diameter of 3.29 ± 0.94 nm, and a crystalline structure consistent with a cubic zinc-blende phase. The incorporation of 5 wt.% CdTe@ZnS shifted the emission of the polymeric system to approximately 635 nm and produced a marked decrease in fluorescence intensity in the presence of oxygen. These changes suggest an interaction between the merocyanine form and metal centers at the quantum-dot surface, which may restrict molecular motion, rigidify the conjugated structure, and modify the excited-state behavior of the organic component. The hybrid system also exhibited greater antibacterial activity against MSSA than the individual components under the evaluated conditions. Therefore, the combined photophysical and biological results indicate an enhanced response upon combining the components and are consistent with the participation of ROS-related photodynamic processes. This hybrid architecture represents a promising approach to developing light-responsive antimicrobial materials by integrating molecular photoswitches, polymeric matrices, and semiconductor quantum dots.

## Figures and Tables

**Figure 1:**
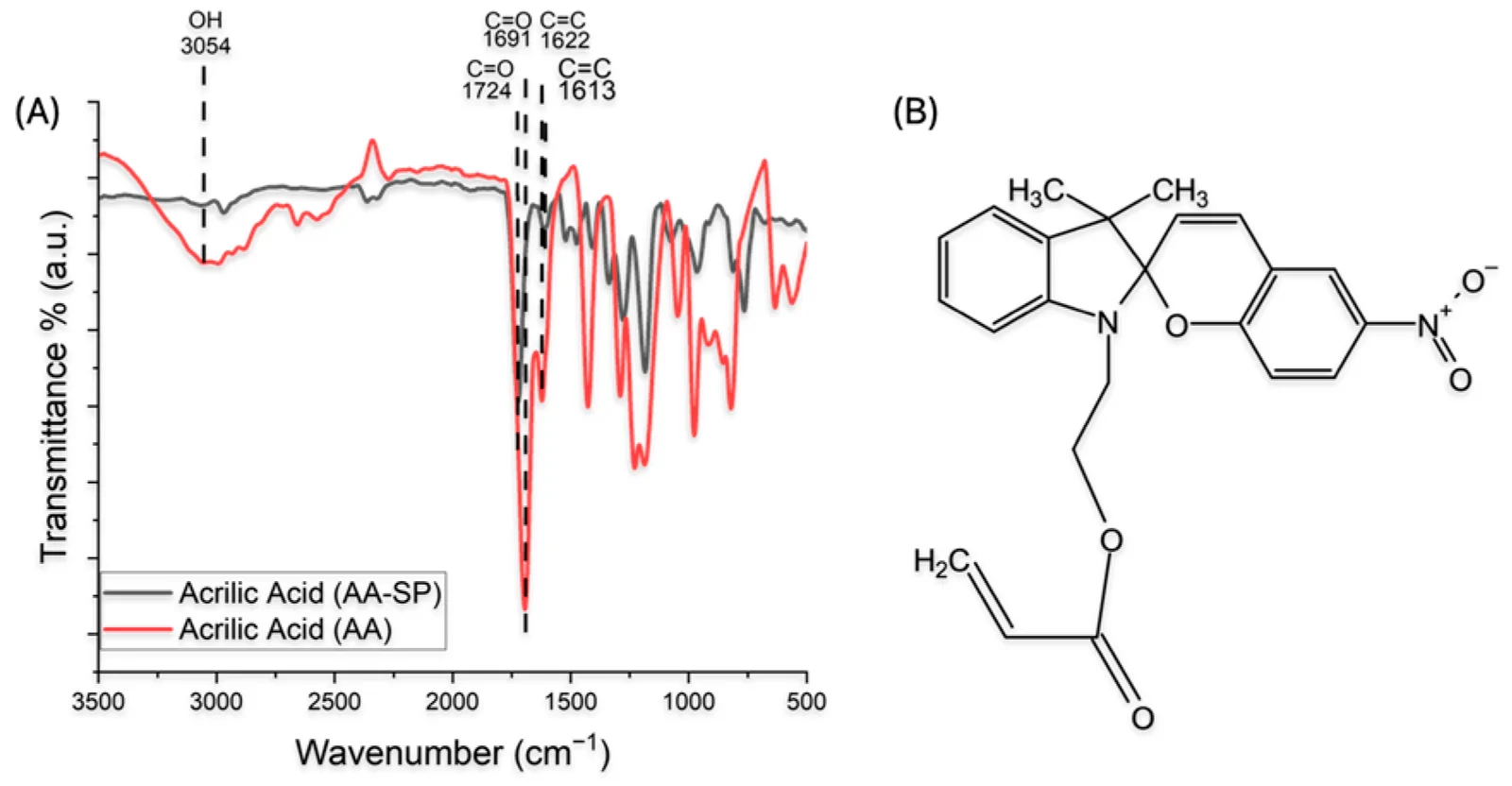
(**A**) FT-IR spectra of acrylic acid AA and spiropyran-functionalized acrylic monomer AA-SP. The comparison highlights the disappearance of the O–H stretching band and the presence of characteristic ester C=O and aromatic C=C bands, confirming successful esterification. (**B**) Chemical structure of the spiropyran-functionalized acrylic monomer AA-SP, highlighting the ester linkage between acrylic acid and the spiropyran moiety, while preserving the polymerizable vinyl group.

**Figure 2:**
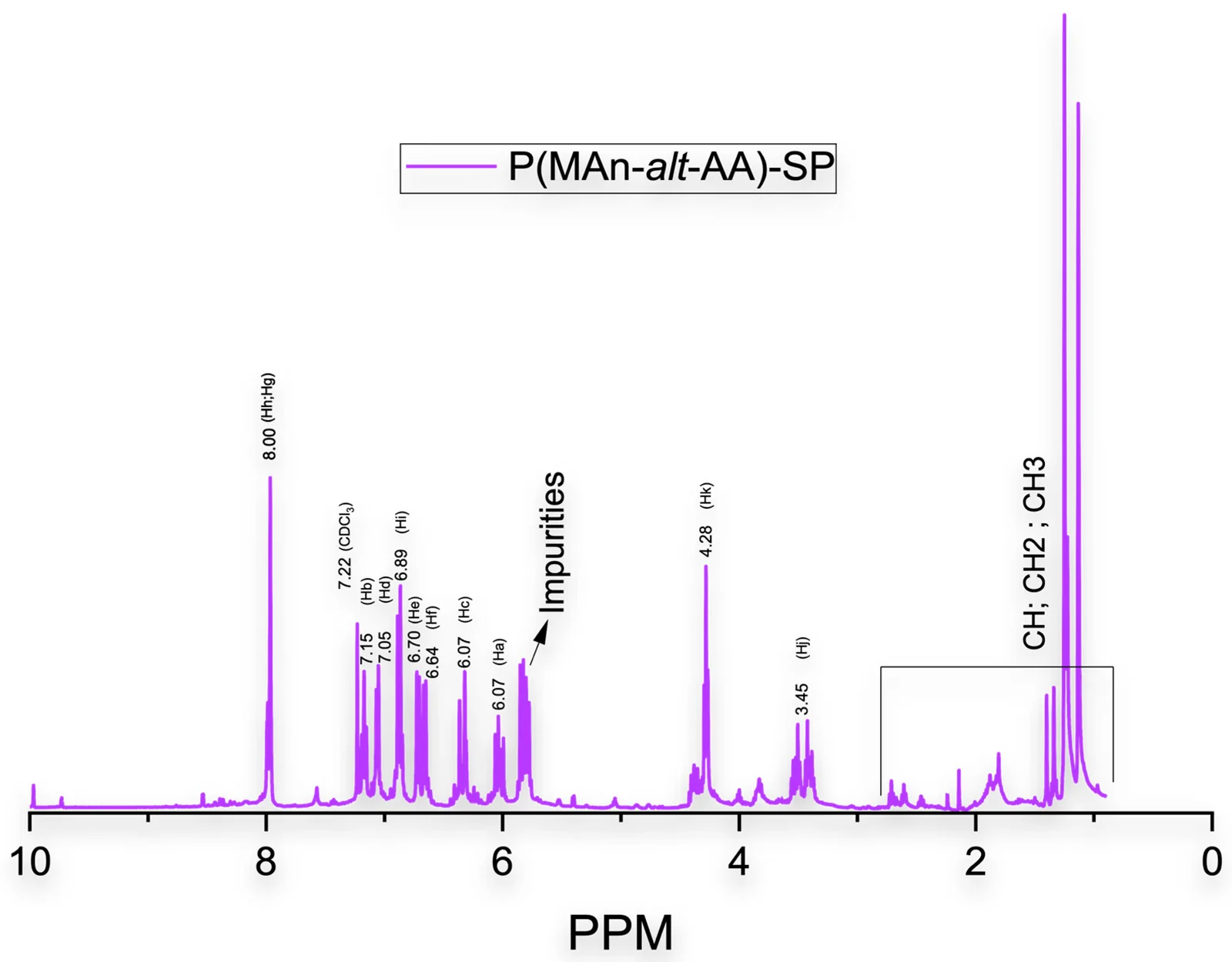
^1^H NMR (δ, ppm, CDCl_3_) spectrum of P(MAn-*alt*-AA)-SP copolymer.

**Figure 3:**
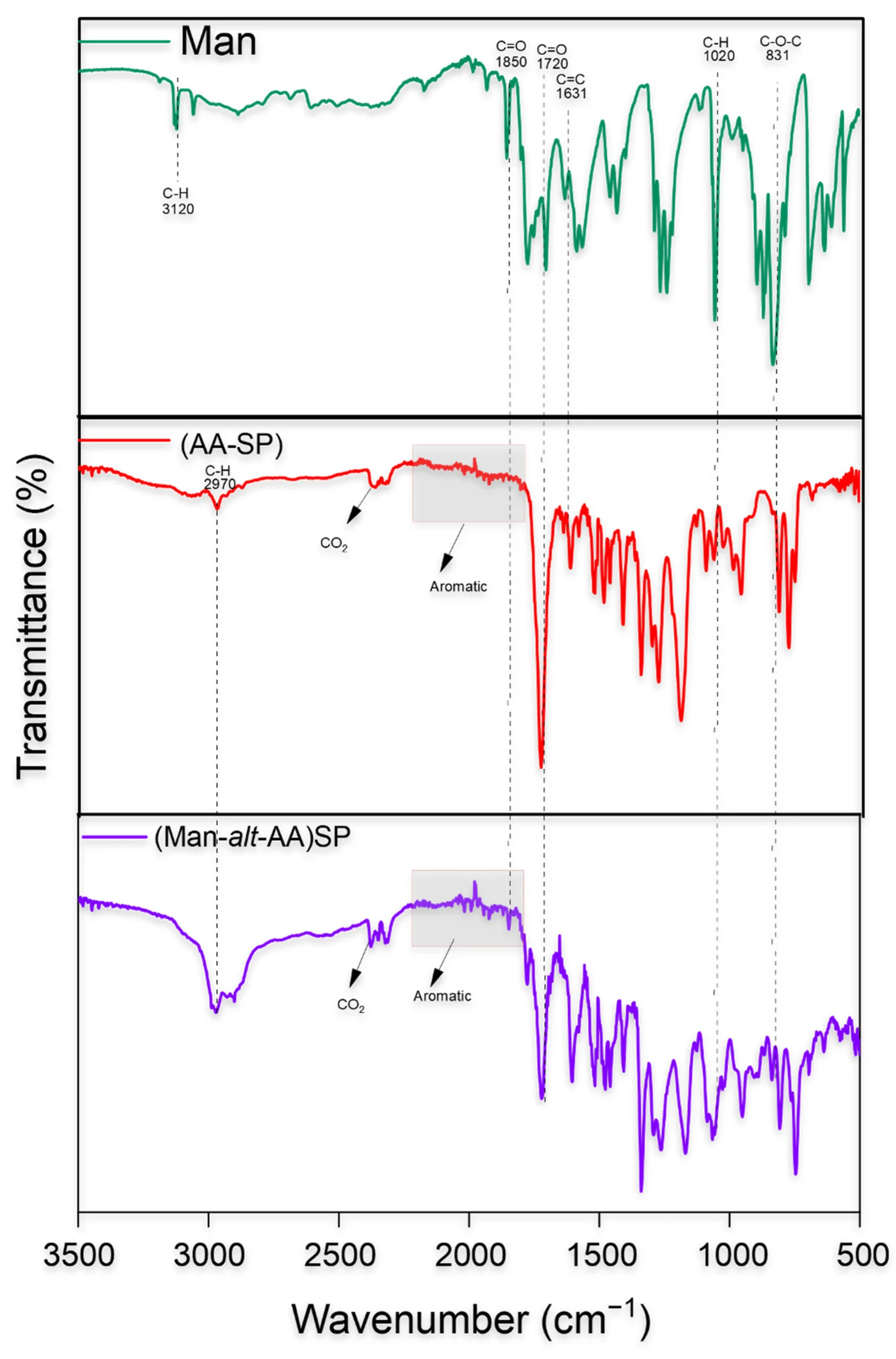
FTIR spectra of maleic anhydride MAn, spiropyran-functionalized acrylic monomer AA-SP, and the resulting P(MAn-*alt*-AA)-SP copolymer, showing the disappearance of vinyl C=C bands and the preservation of anhydride and aromatic functionalities after copolymerization.

**Figure 4:**
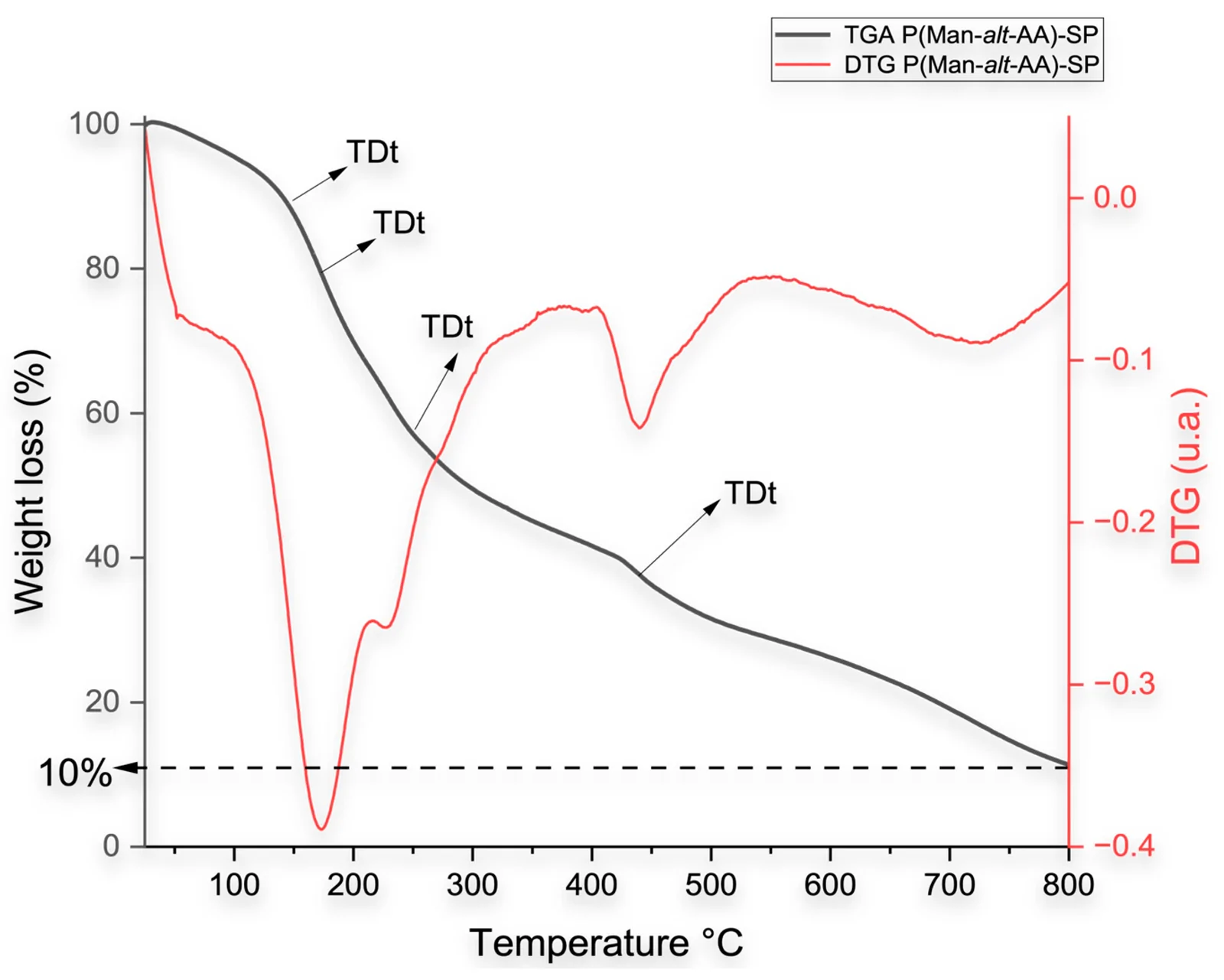
Thermogravimetric analysis of the P(MAn-*alt*-AA)-SP.

**Figure 5:**
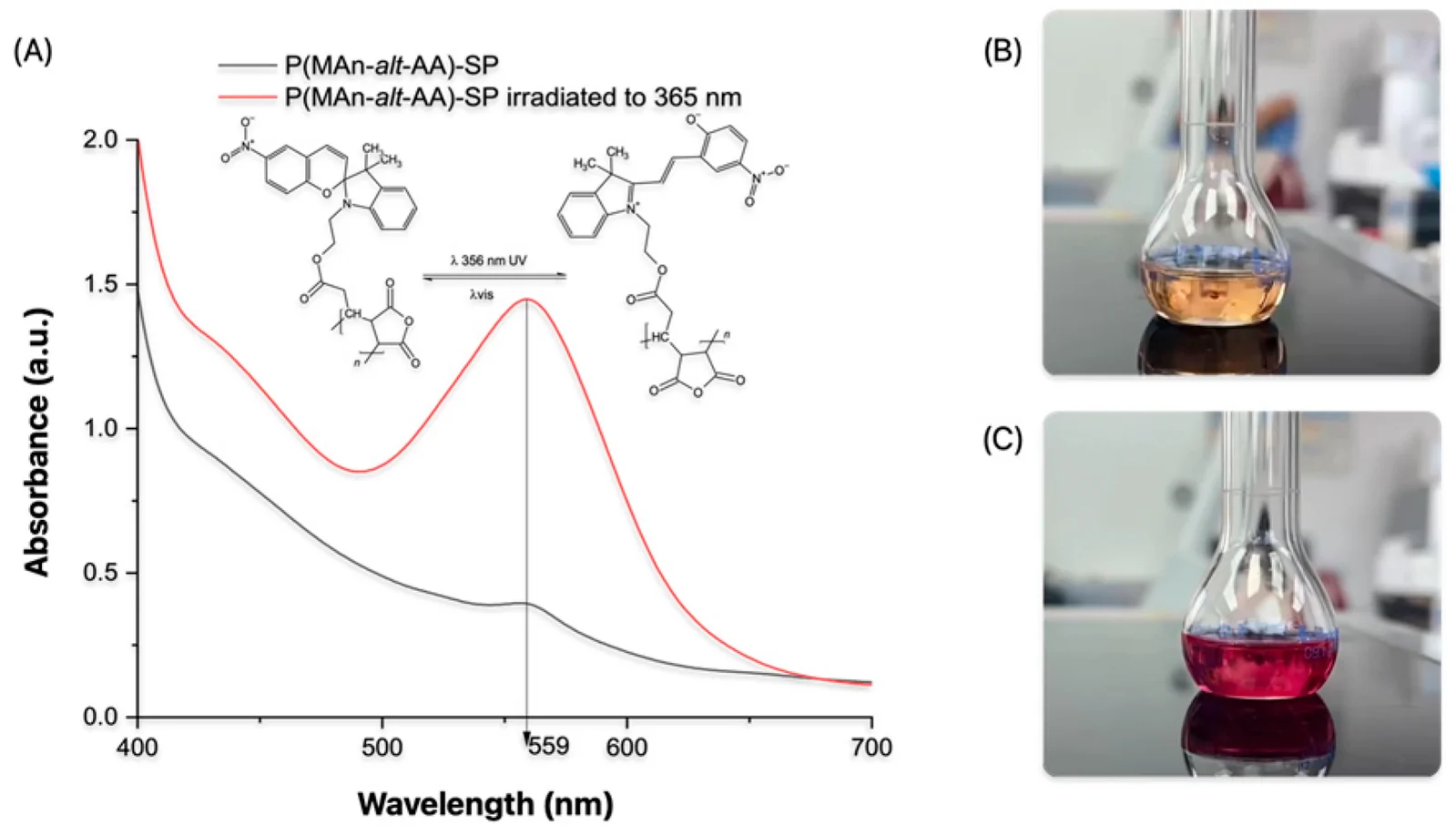
(**A**) UV–Vis absorption spectrum of P(MAn-*alt*-AA)-SP copolymer in chloroform (0.2 mg/mL). (**B**) polymer solution before irradiation. (**C**) polymer solution after irradiation at 365 nm for 5 min.

**Figure 6:**
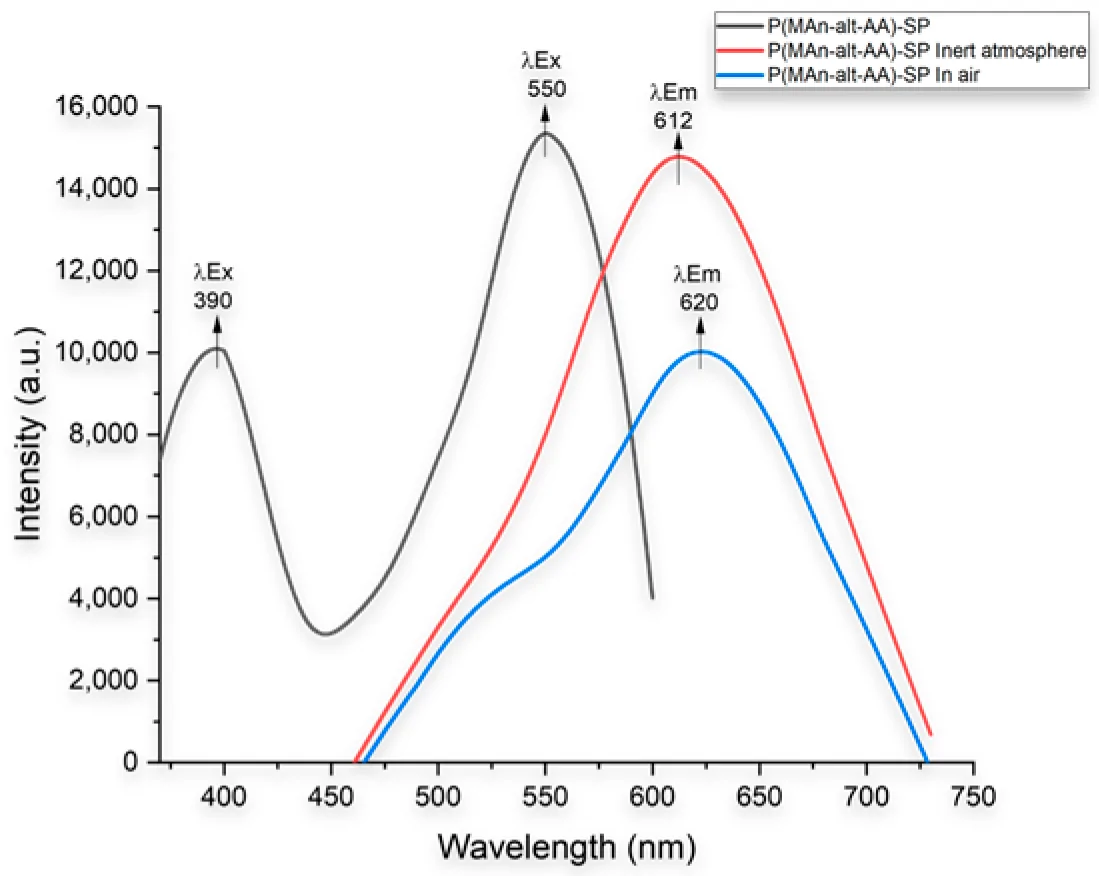
Fluorescence excitation spectrum (λEx) of the P(MAn-*alt*-AA)-SP copolymer (black) and fluorescence emission (λEm) spectra recorded under ambient conditions (blue) and an inert N_2_ atmosphere (red). The emission spectra were recorded using λEx = 390 nm and a slit bandwidth of 10 nm.

**Figure 7:**
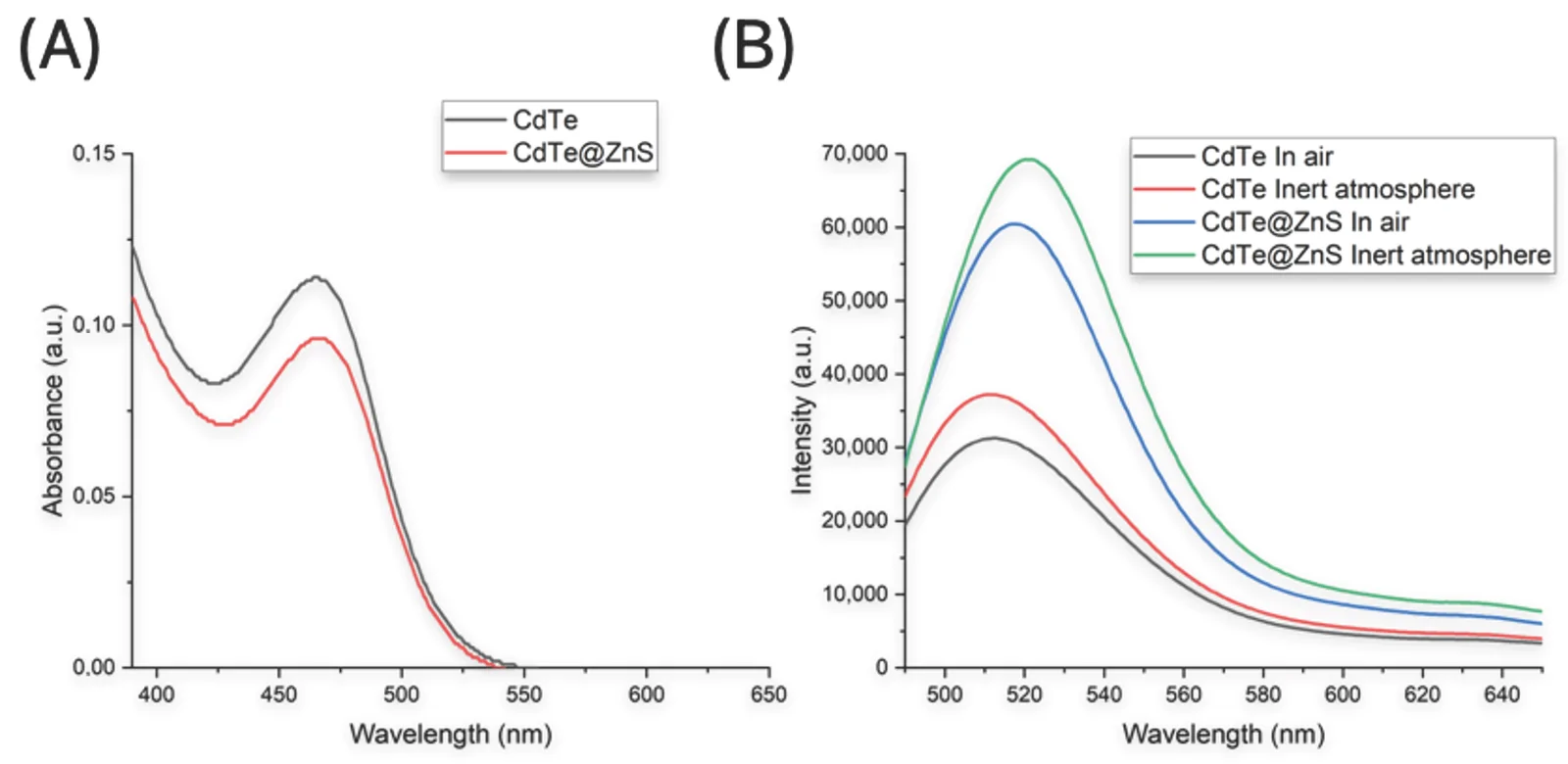
(**A**) UV–Vis absorption spectra of CdTe and CdTe@ZnS quantum dots dispersed in Milli-Q water at 0.03 mg/mL, recorded using quartz UV–Vis cuvettes with a 1 cm optical path length. (**B**) Fluorescence emission spectra of CdTe and CdTe@ZnS quantum dots at the same concentration under ambient and inert N_2_ atmospheres, recorded using quartz fluorescence cuvettes with a 1 cm optical path length.

**Figure 8:**
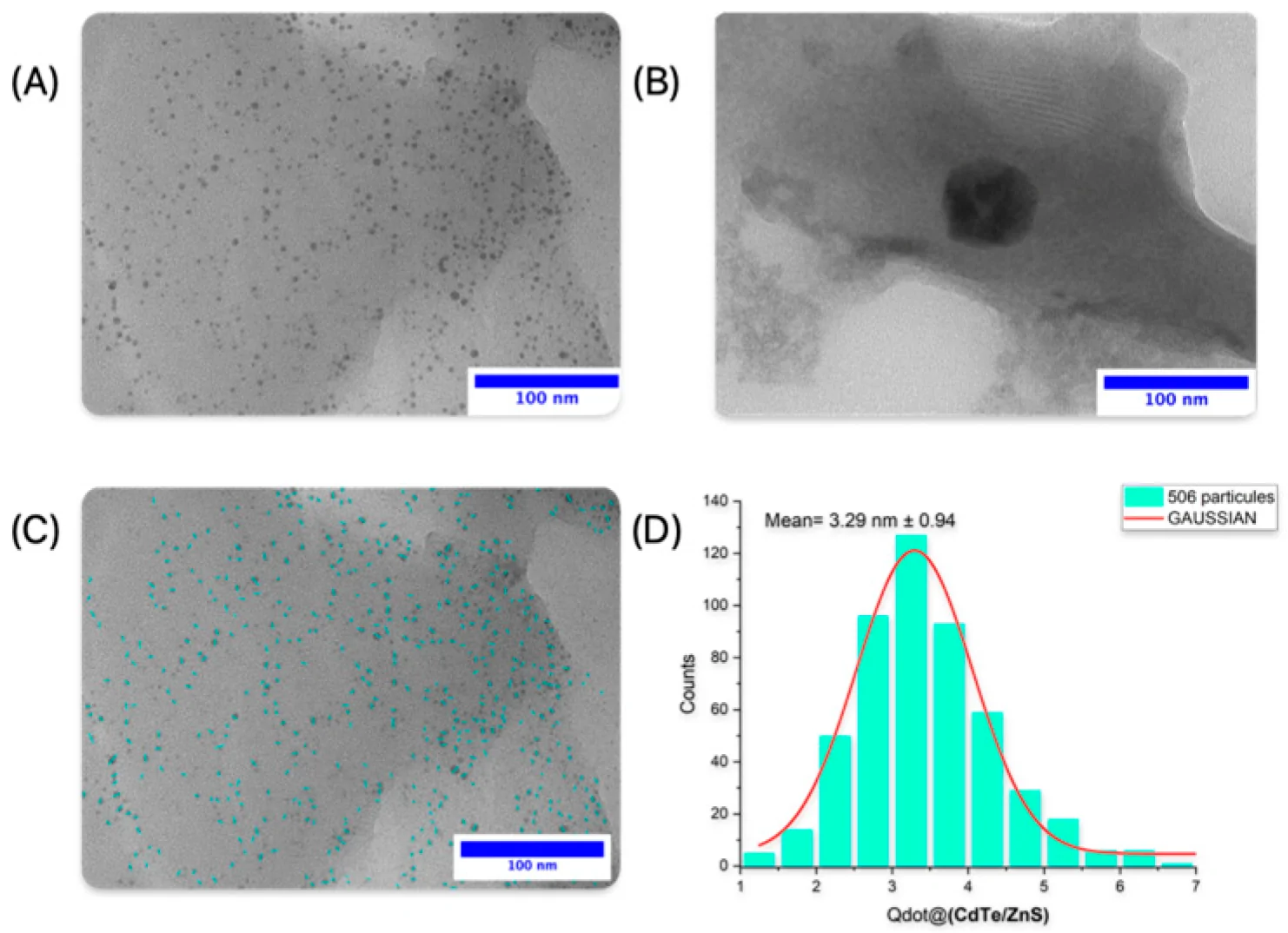
(**A**) Representative TEM micrograph showing the morphology and spatial distribution of the synthesized quantum dots. (**B**) Representative TEM micrograph of the P(MAn-*alt*-AA)-SP/CdTe@ZnS hybrid material, showing a high-contrast inorganic domain associated with the polymeric matrix. (**C**) Processed TEM image used for quantum dot counting, with individual nanoparticles highlighted. (**D**) Particle size distribution histogram obtained from the analysis of 506 nanoparticles, fitted with a Gaussian function (red curve), indicating the average particle size and size dispersion.

**Figure 9:**
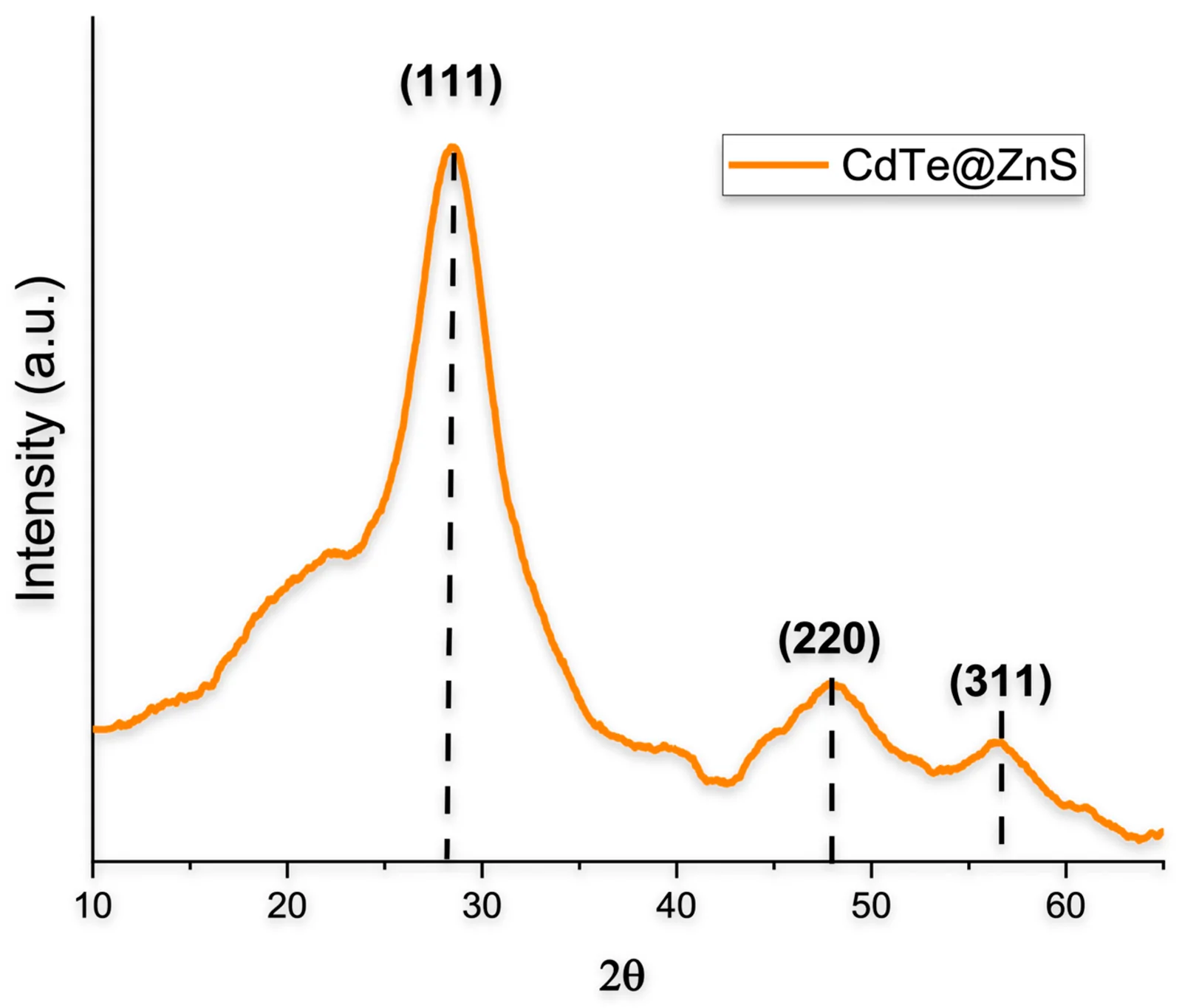
XRD pattern of CdTe@ZnS quantum dots showing characteristic reflections of the cubic zinc blende structure, consistent with core–shell formation.

**Figure 10:**
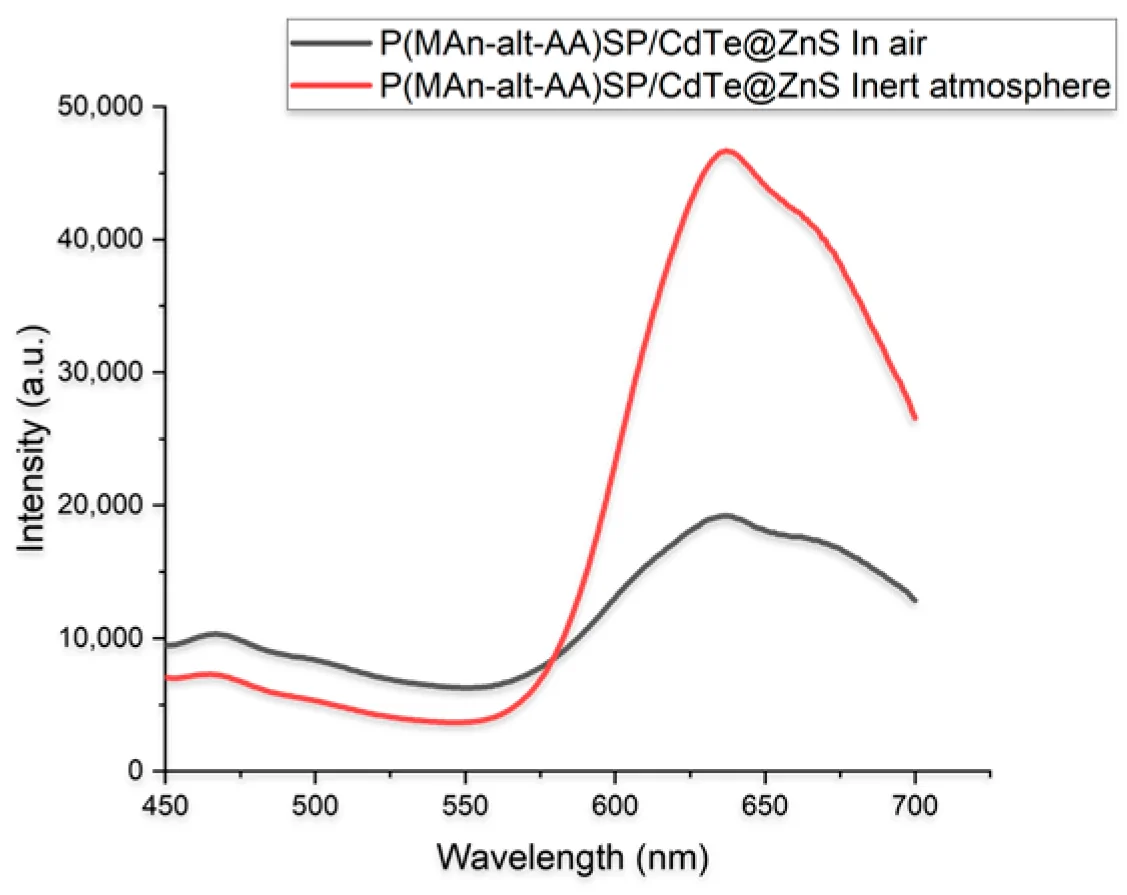
Fluorescence emission spectra of the P(MAn-*alt*-AA)-SP/CdTe@ZnS hybrid system under ambient and inert N_2_ atmospheres. The material was prepared with 5 wt.% CdTe@ZnS relative to the dry mass of the copolymer and analyzed at a copolymer concentration of 0.2 mg/mL using an excitation wavelength of 390 nm and a slit bandwidth of 10 nm.

**Figure 11:**
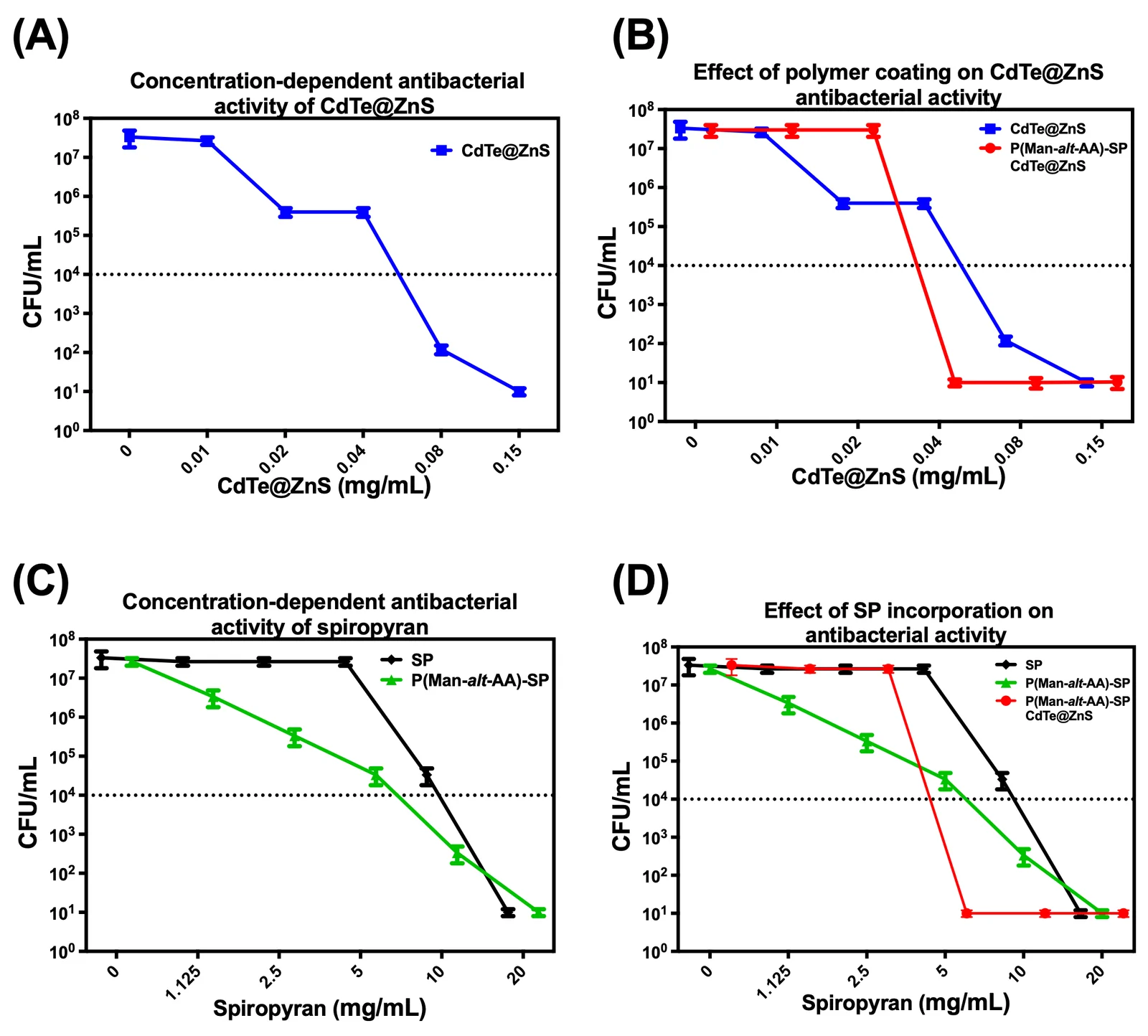
Concentration-dependent antibacterial activity of CdTe@ZnS, spiropyran SP, and their polymer-coated formulations. (**A**) Effect of CdTe@ZnS concentration on bacterial viability. (**B**) Effect of polymer coating with P(MAn-*alt*-AA)-SP on the antibacterial activity of CdTe@ZnS. (**C**) Concentration-dependent antibacterial activity of free SP and P(MAn-*alt*-AA)-SP. (**D**) Effect of SP incorporation into P(MAn-*alt*-AA)-SP conjugated with a fixed CdTe@ZnS concentration at 20 µg/mL on bacterial viability. Bacterial growth is expressed as the mean ± SD of CFU/mL.

**Figure 12:**
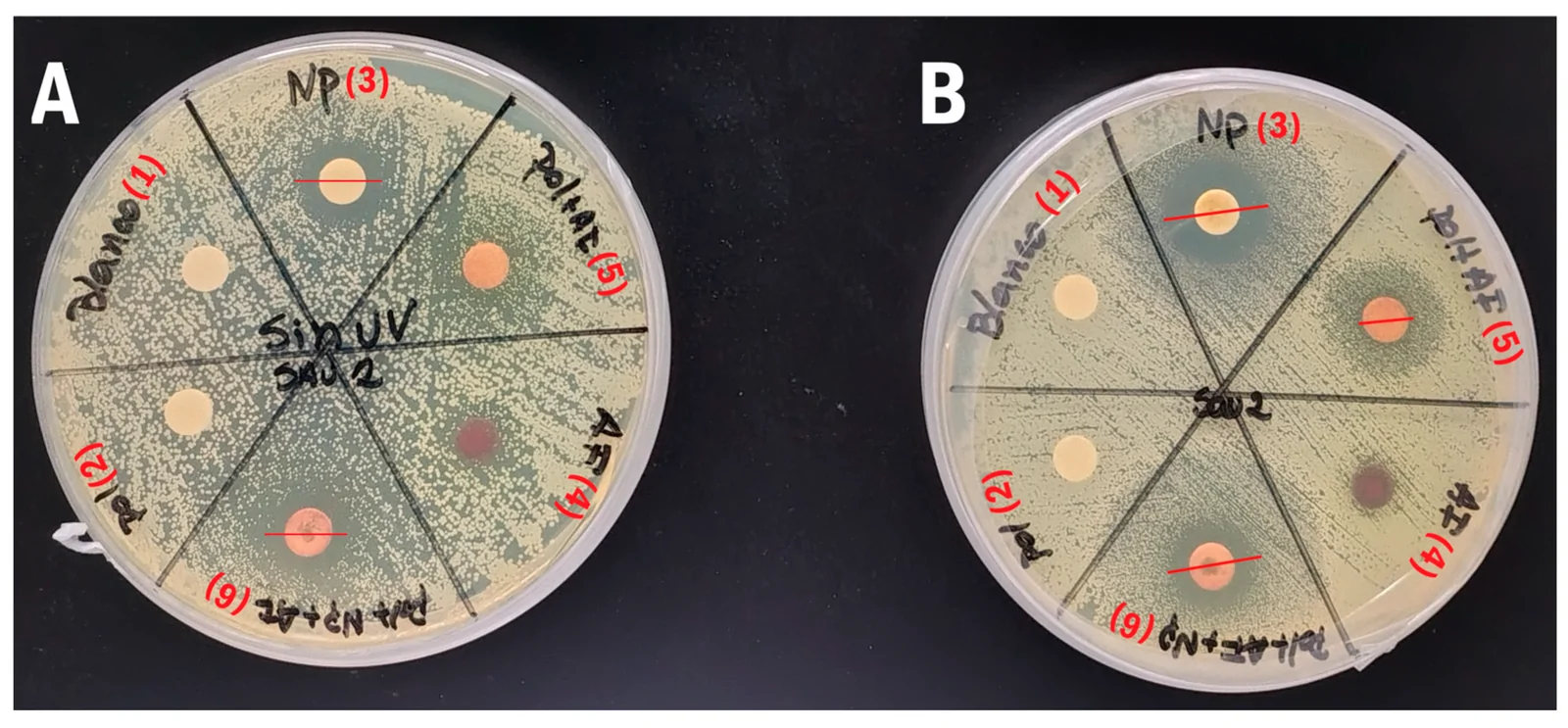
Comparison of inhibition zone diameters under irradiated (**A**) and non-irradiated (**B**) conditions for the different formulations against MSSA: (1) Blank (negative control), (2) P(MAn-*alt*-AA), (3) CdTe@ZnS, (4) Spiropyran SP, (5) P(MAn-*alt*-AA)-SP, and (6) P(MAn-*alt*-AA)-SP/CdTe@ZnS. Inhibition zones are clearly delineated, and data represent the mean ± SD (n = 3).

**Table 1:** Inhibition zone diameters obtained by the Kirby–Bauer agar diffusion assay against MSSA under irradiated and non-irradiated conditions (Table S1 *n* = 3).

Treatment (Plate Sector)	Inhibition Zone Diameter (mm) with Irradiation (365 nm + 525 nm)	Inhibition Zone Diameter (mm) Without Irradiation
(1) Blank (negative control)	0.0 ± 0.00	0.0 ± 0.00
(2) P(MAn-*alt*-AA)	0.0 ± 0.00	0.0 ± 0.00
(3) CdTe@ZnS	10.7 ± 7.11	4.2 ± 5.56
(4) Spiropyran SP	8.7 ± 2.44	0.0 ± 0.00
(5) P(MAn-*alt*-AA)-SP	14.5 ± 1.33	10.0 ± 0.67
(6) P(MAn-*alt*-AA)-SP/CdTe@ZnS	14.9 ± 0.47	10.7 ± 1.11

## Data Availability

The data presented in this study are available on request from the corresponding author. The data are subject to institutional protection policies and are therefore not publicly available. However, they will be made available by the corresponding author upon reasonable request.

## Supplementary Materials

The following supporting information can be downloaded at: https://www.mdpi.com/article/10.3390/polym18202478/s1, Figure S1: ^1^H NMR spectrum (400 MHz, CDCl_3_) of the spiropyran-functionalized acrylic monomer AA-SP; Figure S2: Two-dimensional ^1^H–^1^H COSY spectrum of the spiropyran-functionalized acrylic monomer AA-SP recorded in CDCl_3_; Figure S3: Original TEM micrograph of the synthesized CdTe@ZnS quantum dots, showing their morphology and dispersion; Figure S4: Original TEM micrograph of the P(MAn-*alt*-AA)-SP/CdTe@ZnS hybrid material, showing the inorganic component associated with the polymeric matrix; Table S1: Inhibition zone diameters obtained by the Kirby–Bauer agar diffusion assay against MSSA under irradiated and non-irradiated conditions (*n* = 3, mean ± SD).
