# Supplementary material for: New Smart Polymeric Materials with Optical Sensing Properties: Determination of Antimicrobial Properties Using Photodynamic Therapy
Source: Polymers (Basel). 2026 Oct 10;18(20):2478. doi: 10.3390/polym18202478 (PMC13708952; doi:10.3390/polym18202478)
Supplement: Supplementary file 1 [file polymers-18-02478-s001.zip › polymers-4561462-supplementary.pdf]

Supplementary Materials

3. Results

3.1. Characterization of the Spiropyran-Functionalized Acrylic Monomer (AA–SP)

1H NMR characterization of AA-SP. The structure of the spiropyran-functionalized acrylic monomer AA-SP was further evaluated by ^{1}H NMR and two-dimensional ^{1}H–^{1}H COSY spectroscopy in deuterated chloroform ($CDCl_3$). The ^{1}H NMR spectrum shows resonances in the aromatic and olefinic region between approximately δ 5.8 and 8.0 ppm, consistent with the proton environments of the spiropyran and acrylate moieties. The signals at approximately δ 6.38, 6.06, and 5.83 ppm correspond to the vinyl protons of the acrylate group, supporting preservation of the polymerizable C=C bond after functionalization. Signals at approximately δ 4.4 and 2.7 ppm are attributed to the methylene proton environments of the –N–CH_2–CH_2–O– linkage, while resonances in the approximately δ 1.1–1.4 ppm region are associated with the methyl groups of the spiropyran unit. Importantly, no broad resonance is observed in the approximately δ 11–12 ppm region characteristic of the carboxylic acid proton of acrylic acid, which is consistent with ester formation. The 2D COSY spectrum shows proton–proton correlations within the vinyl/aromatic region and a correlation between the methylene proton environments at approximately δ 4.4 and 2.7 ppm, supporting their assignment to the same –N–CH_2–CH_2–O– fragment. Together with the FTIR results, these NMR data provide complementary evidence for the successful formation of the AA-SP monomer.

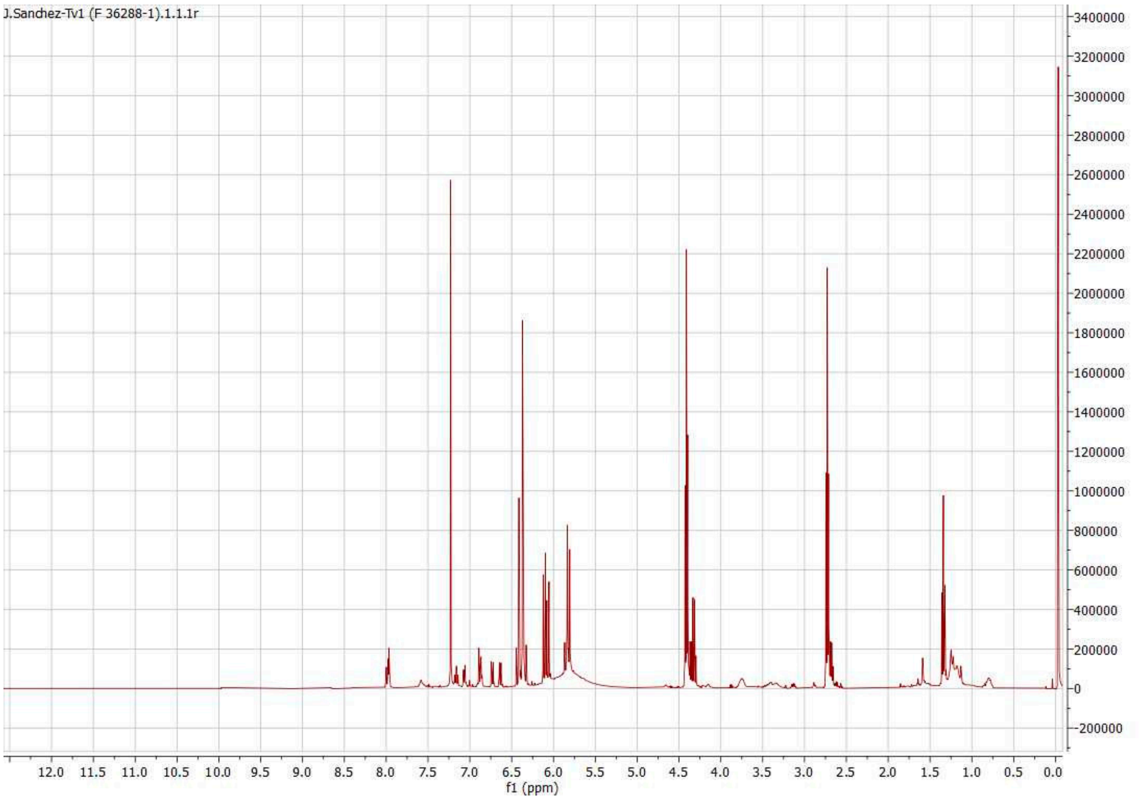

Figure S1. ^{1}H NMR spectrum (400 MHz, $CDCl_3$) of the spiropyran-functionalized acrylic monomer AA-SP.

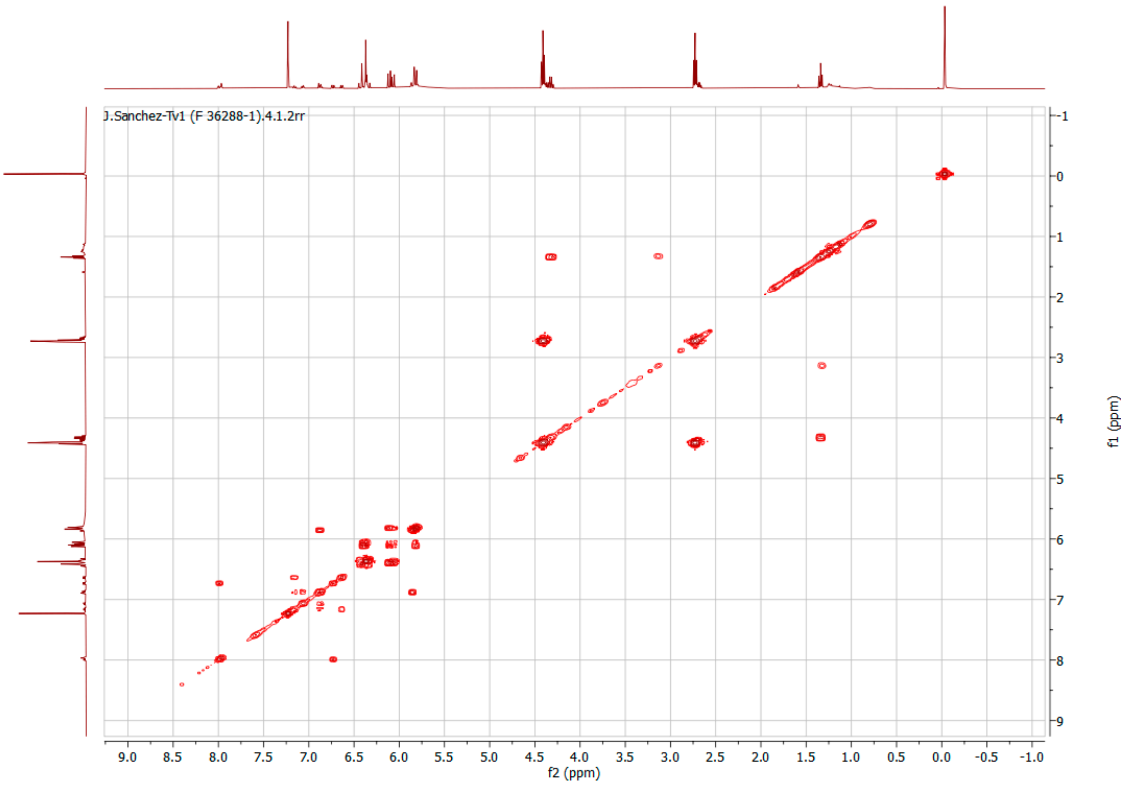

Figure S2. Two-dimensional ^{1}H–^{1}H COSY spectrum of the spiropyran-functionalized acrylic monomer AA-SP recorded in $CDCl_3$.

3.4. Physical Characterization of CdTe@ZnS Quantum Dots and Hybrid Material

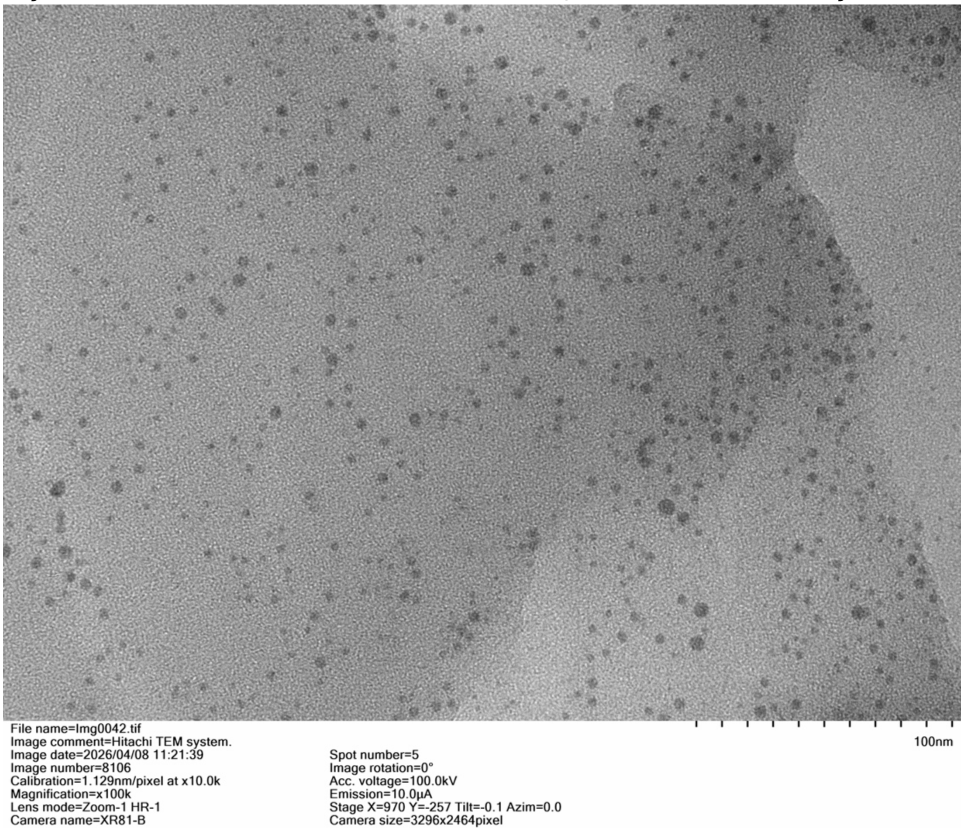

Figure S3. Original TEM micrograph of the synthesized CdTe@ZnS quantum dots, showing their morphology and dispersion.

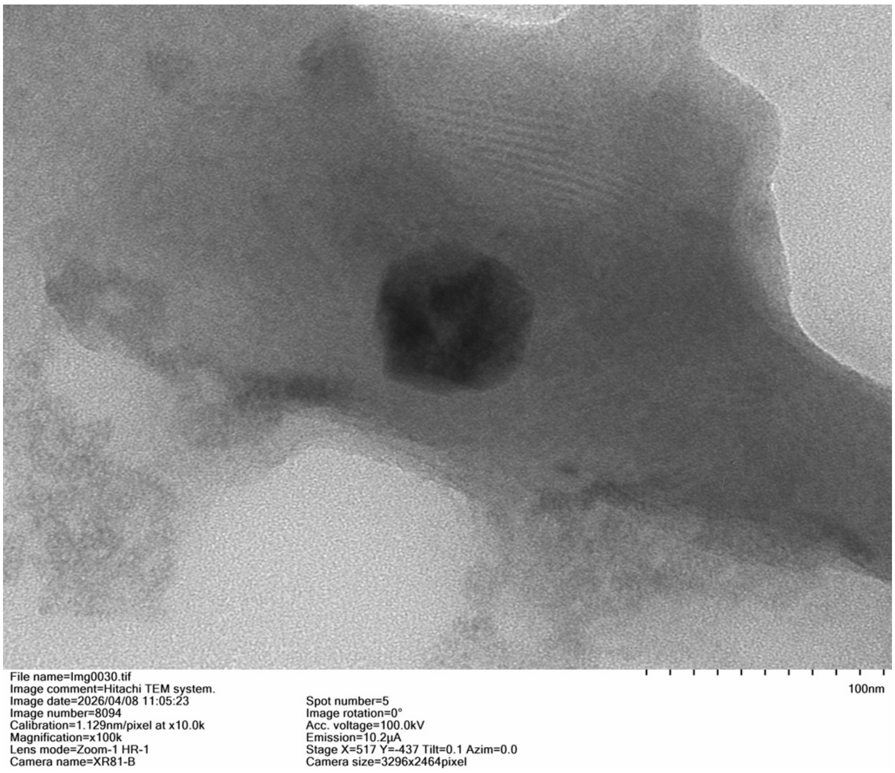

Figure S4. Original TEM micrograph of the P(MAn-alt-AA)-SP/CdTe@ZnS hybrid material, showing the inorganic component associated with the polymeric matrix.

3.7 Antimicrobial activity evaluated by the Kirby–Bauer agar diffusion assay

Table S1. Inhibition zone diameters obtained by the Kirby–Bauer agar diffusion assay against MSSA under irradiated and non-irradiated conditions (n = 3, mean ± SD).

Treatment (plate sector)	Condition	MSSA 1	MSSA 2	MSSA 3	Mean	SD
Blank (negative control)	Irradiated	0.00	0.00	0.00	0.00	0.00
	Non-irradiated	0.00	0.00	0.00	0.00	0.00
P(MAn-*alt*-AA)	Irradiated	0.00	0.00	0.00	0.00	0.00
	Non-irradiated	0.00	0.00	0.00	0.00	0.00
CdTe@ZnS	Irradiated	15.00	17.00	0.00	10.67	7.11
	Non-irradiated	12.50	0.00	0.00	4.17	5.56
Spiropyran (SP)	Irradiated	11.50	10.00	5.00	8.66	2.44
	Non-irradiated	0.00	0.00	0.00	0.00	0.00
P(MAn-*alt*-AA)-SP	Irradiated	16.50	14.50	12.50	14.50	1.33
	Non-irradiated	11.00	10.00	9.00	10.00	0.67
P(MAn-alt-AA)-SP/CdTe@ZnS	Irradiated	15.00	15.50	14.20	14.90	0.47
	Non-irradiated	12.00	11.00	9.00	10.67	1.11
